# Comprehensive Transcriptome and Proteome Analyses Reveal the Modulation of Aflatoxin Production by *Aspergillus flavus* on Different Crop Substrates

**DOI:** 10.3389/fmicb.2020.01497

**Published:** 2020-07-14

**Authors:** Xu Li, Yiran Jiang, Longxue Ma, Xiaoyun Ma, Yang Liu, Jihao Shan, Kang Ma, Fuguo Xing

**Affiliations:** ^1^Key Laboratory of Agro-Products Quality and Safety Control in Storage and Transport Process, Ministry of Agriculture and Rural Affairs/Institute of Food Science and Technology, Chinese Academy of Agricultural Sciences, Beijing, China; ^2^School of Food Science and Engineering, Foshan University, Foshan, China; ^3^Division of Chemical Metrology and Analytical Science, National Institute of Metrology, Beijing, China

**Keywords:** food safety, aflatoxin, *Aspergillus flavus*, crop substrate, regulation mechanism

## Abstract

As a natural severe contaminant of stored grains and other crops worldwide, *Aspergillus flavus* can produce aflatoxins (AFs), the most powerful naturally producing toxic and hepatocarcinogenic compounds. AFs production is regulated by diverse factors including AFs cluster genes, transcription factors, regulators, and environmental factors. Among them, crop substrate is one of the most important factors. Here, we found that AFB_1_ production was significantly higher in maize and rice broth than in peanut broth. To clarify the mechanisms involved, complementary transcriptomic and proteomic analyses were performed to identify changes in *A. flavus* incubated in the three crop substrates. The results indicated that fewer genes and proteins were differentially expressed between maize and rice substrates, whereas more differentially expressed genes were observed between maize/rice broth and peanut broth. In particular, the genes involved in the initial step of AFs biosynthesis (*aflA*, *aflB*, and *aflC*) and the ACCase-encoding gene *accA* were significantly upregulated on the maize and rice substrates. Kyoto Encyclopedia of Genes and Genomes (KEGG) and Gene Ontology (GO) enrichment analyses indicated that carbon-metabolism-related genes were obviously enriched in the maize broth, and the genes involved in acetyl-CoA accumulation and consumption were up- and downregulated, respectively. Several genes involved in the regulation of AFs biosynthesis, including *veA*, *ppoB*, *snf1*, and the G-protein-coupled receptor (GPCR) genes, were differentially expressed on the three substrates, suggesting that these genes may be also involved in sugar signal sensing, transfer, and regulation. Interestingly, by the correlation analyses of transcriptome and proteome, trehalose metabolism genes, aldehyde dehydrogenase gene, and tryptophan synthase gene were found to be relevant with the regulation of AFs production on different crop substrates. Taken together, the differential expressions of the AFs cluster genes, several regulatory genes, and carbon metabolism genes were involved in the comprehensive modulation of AFs production on different crop substrates.

## Introduction

*Aspergillus flavus* is widely distributed in tropical and subtropical regions and infects various crops, including wheat, maize, and peanuts. It can produce different kinds of mycotoxins, especially aflatoxins (AFs), which cause huge losses in crop quality, safety, and commodity price (Amaike and Keller, 2011). AFs mainly include four types in crops, AFB_1_, AFB_2_, AFG_1_, and AFG_2_; among them, AFB_1_ is predominant and the most toxic and carcinogenic naturally compound (Marchese et al., 2018). Many studies have shown that the consumption of AF-contaminated food severely impairs human and animal health, especially causing liver cancer (Amaike and Keller, 2011; Marchese et al., 2018; Wang et al., 2019). Therefore, deciphering the regulatory mechanisms of AFs synthesis is extremely essential for us to control AFs contamination.

Aflatoxins biosynthesis has been extensively studied in recent years. In *A. flavus*, a 75-kb gene cluster including 29 genes is involved in AFs biosynthesis (Cleveland et al., 2009). The precursors of AFs, acetyl-CoA, and malonyl-CoA derived from carbon and lipid metabolism, are cyclized by the polyketide synthase AflC, and then a series of enzymatic reactions are performed to generate AFs (Georgianna and Payne, 2009). Among these genes, *aflR* and *aflS* are the two key pathway-specific regulatory genes for AFs biosynthesis (Yin and Keller, 2011). The deletion of *aflR* abolished the expression of all AFs structural genes in the cluster (Woloshuk et al., 1994), and the mutation of *aflS*, encoding a transcription enhancer, caused a significant reduction in AFs production (El Khoury et al., 2011). In addition, the ratio of *aflS* and *aflR* is an indicator of the AFs biosynthesis activation (Schmidt-Heydt et al., 2010).

As secondary metabolites, AFs production is also regulated by various factors with complicated mechanisms. CreA, the central regulator of carbon catabolite repression, strongly affects AFs production (Fasoyin et al., 2018). The nitrogen regulator AreA binds to the intergenic region between *aflR* and *aflS*, thus disturbing the transcription of the AFs cluster (Morozov et al., 2001; Ehrlich et al., 2003). The modulation of AFs biosynthesis under acidic and alkaline conditions is associated with the pH regulator PacC (Ehrlich et al., 2003; Bignell et al., 2005). VeA physically interacts with VelB and LaeA to form the velvet regulatory complex, which modulates light-responsive development and the production of secondary metabolites (Duran et al., 2007; Amaike and Keller, 2009; Lv Y. et al., 2018). Oxidative-stress-related transcription factors (Ap-1, SrrA, AtfB, MsnA, etc.) are activated by the stress signal transduction pathway, such as the mitogen-activated protein kinase (MAPK) pathway (Ren et al., 2016), and most of them affect AFs synthesis in *A. flavus* (Reverberi et al., 2005). Fungal-development-related genes, such as *fadA*, *fluG*, *ppoABC*, and the G-protein-coupled receptor (GPCR) genes, are also involved in the modulation of AFs synthesis (Amare and Keller, 2014). Although AFs regulatory network has been reported, many regulation mechanisms and regulators are still undiscovered.

The different components of diverse crop substrates significantly influence fungal colonization, development, and secondary metabolite production (Palencia et al., 2010; Korani et al., 2017). *A*. *flavus* contaminates a great variety of agricultural foods, including not only cereals such as maize, wheat, and rice but also oil-rich nuts, such as peanut, sunflower seed, and almond. AFs contamination levels significantly depend on the different crops under artificial inoculation and natural occurrence (Iqbal et al., 2006; Njumbe et al., 2014). However, little information has focused on the regulatory mechanisms of AFs biosynthesis in different crops. In this study, to mimic the AFs production by *A. flavus* in different crops, artificial media were prepared using maize, rice, and peanut. Transcriptomic and proteomic analyses were performed to identify the genes or pathways involved in the regulation of AFs biosynthesis on different crop substrates.

## Materials and Methods

### Strain and the Preparation of Culture Media

*Aspergillus flavus* strain YC-15 was isolated in Hubei Province (Liu et al., 2017) and maintained on potato dextrose agar (PDA) medium. To prepare the 10^9^ conidia/ml suspension, the strain was cultured for 7 days, and the conidia were collected with 0.1% Tween 20 solution and counted with a hemocytometer.

The yeast extract sucrose (YES) broth was performed as before (Bai et al., 2015). The crop substrates were prepared as follows: 100 g crop samples (maize, rice, and peanut) were carefully milled to powder by the grinder (WZDC, Beijing, China), boiled with 800 ml H_2_O for 20 min, filtered with a treble-layer cheesecloth, and added H_2_O up to 1 L. Each 200 ml medium was placed in a 500-ml flask and autoclaved at 121°C for 20 min.

### Determination of Mycelial Dry Weight and AFB_1_ Production

Conidia suspension was inoculated into diverse media at a final concentration of 1 × 10^6^ conidia/ml. All treatment groups were prepared in triplicate and cultured in the dark at 28°C with 200 rpm. After 7 days cultivation, the fermentation solutions were filtered with filter paper, and the mycelia were weighted after drying for 12 h at 60°C. AFB_1_ was extracted according to the method described by Liang et al. (2015) with minor modification. The 25-ml supernatant was mixed and extracted with 25 ml chloroform, evaporated under N_2_ flow, and redissolved by 10 ml methanol. The 10 ml extract solution was purified using the ToxinFast immunoaffinity column (Huaan Magnech Bio-tech, Beijing, China) for AFB_1_ detection. AFB_1_ was detected by Agilent 1220 Infinity II with a fluorescence detector (Agilent, Santa Clara, CA, United States), a postcolumn derivatization system (Huaan Magnech Bio-tech, Beijing, China), and an Agilent TC-C18 column (250 mm × 4.6 mm, 5 μm particle size, Agilent).

### RNA Extraction and Illumina Sequencing

RNA extraction and Illumina sequencing were performed according to Lv C. et al. (2018). Mycelia were filtered and harvested after 7 days. Then, the mycelia were frozen in liquid nitrogen and grounded to fine powder. Total RNA was extracted with TRIzol Reagent (Invitrogen, Shanghai, China) according to the instructions, and genomic DNA was digested using DNase I. The RNA quality was evaluated with NanoDrop 2000 spectrophotometer (Thermo Fisher, Waltham, MA, United States) and Agilent 2100 Bioanalyzer (Agilent, Santa Clara, CA, United States).

RNA-Seq experiments were performed by Novogene (Beijing, China). Briefly, messenger RNA (mRNA) was purified from total RNA with oligo-dT magnetic beads. Sequencing libraries were generated with NEB Next^®^ Ultra^TM^ RNA Library Prep Kit for Illumina^®^ (NEB, United States), and non-strand-specific libraries were sequenced on an Illumina HiSeq 4000 platform (Illumina Inc., San Diego, CA, United States). The 150-bp paired-end reads were generated. Raw data were submitted to the National Center for Biotechnology information (NCBI) Sequence Read Archive under the accession number of PRJNA605915.

### Transcriptomic Data Processing

Raw reads were filtered out with NGQC software (Novogene, Beijing, China). Raw data were trimmed to remove adaptor sequence, reads with adaptor contaminants, low-quality reads (the number of bases with *Q* value ≤ 20 accounts for more than 50%), and reads with more than 10% N (N indicates that base information that cannot be determined). Then, clean reads were mapped to the *A. flavus* NRRL3357 genome database (BioProject: PRJNA13284) using the HISAT 1.31 with recommended parameters (Kim et al., 2015).

The expression levels were calculated with read counts. Read counts normalization was performed with DESeq R package (1.10.1). Because of the experiment with biological replicates in this study, DESeq method was used for read count normalization. The *p* values were used to identify the differentially expressed genes (DEGs) of *A. flavus* YC-15 in different media (Trapnell et al., 2010). *p* value was calculated by the negative binomial distribution, and false discovery rate (FDR) was counted by Benjamini–Hochberg method for *p* values correction. The adjusted *p* value (*p*_adj_) < 0.05 and fold change (FC) > 1.5 were set as the threshold for DEG. Finally, the DEGs were subjected to Gene Ontology (GO) functional analysis and Kyoto Encyclopedia of Genes and Genomes (KEGG) pathway enrichment with FungiFun and KAAS, respectively (Kanehisa et al., 2008; Priebe et al., 2011).

### Protein Preparation and Liquid Chromatography–Tandem Mass Spectroscopy Analysis

Protein preparation and liquid chromatography–tandem mass spectroscopy (LC-MS/MS) procedures were based on the methods reported by Bai et al. (2015) and Lu et al. (2010). Different broths were inoculated with *A. flavus* conidia and cultured at 28°C for 7 days. To extract intracellular proteins, mycelia were harvested by centrifugation at 12,000 rpm for 15 min and washed twice with phosphate-buffered saline (PBS) to remove any extracellular protein. The pellet was resuspended in lysis buffer [SDT solution: 4% sodium dodecyl sulfate (SDS), 100 mM dithiothreitol (DTT), 150 mM Tris–HCl, pH 8.0], sonicated on ice (80 W, 10 s, 10 times), centrifuged to remove the insoluble component, and stored at −80°C. Extracellular proteins of fermentation were harvested by removing the biomass and filtering the supernatant through a 0.2-μm filter. Supernatant was mixed the equivoluminal SDT solution, and other steps were the same as those described above. Three biological repeats of both intra- and extracellular proteins were extracted from four different media.

All protein samples were digested with trypsin (Promega, Madison, WI, United States) overnight at 37°C and collected by centrifugation. After desalting and acidification, the resulting peptide mixture was labeled with 8-Plex iTRAQ Reagents according to the manufacturer’s instructions (Applied Biosystems, Forest City, CA, United States). High-pH reverse-phase fractionation was performed to fractionate the labeled digested samples into fractions by increasing acetonitrile step-gradient elution. An LC-MS/MS analysis was performed on a Q Exactive Mass Spectrometer coupled to a nanoflow high-performance liquid chromatography (HPLC) apparatus (Thermo Fisher, Waltham, MA, United States). The peptide mixture was injected into the Easy-Spray C18-reversed phase column (Thermo Fisher) in buffer A (0.1% formic acid) and separated with a linear gradient of buffer B (80% acetonitrile and 0.1% formic acid) at a flowrate of 250 nl/min. The MS data were acquired with a 2.5-kV ion spray voltage, 30 psi curtain gas, 15 psi nebulizer gas, and the 150°C interface temperature. MS data were acquired with >30,000 full-width reversed phase scanning, and a data-dependent top 10 method was used to choose the most abundant precursor ions from the survey scan (300–1,800 m/z).

### Proteomic Data Processing

After raw data collection, the MaxQuant software (version: 1.5.3.17)^1^ was used to process the mass data with the recommended parameters (Cox and Mann, 2008). Two missed cleavages were allowed for tryptic peptides, the mass tolerance was set to 20 ppm, and the modification pattern was added including carbamidomethyl, oxidation, acetyl, etc. Protein annotation was performed by searching the *A. flavus* database (uniprot_Aspergillus_flavus_13501_20171127.fasta)^2^, and the database pattern was set to the Target-Reverse. At least two peptides per protein, and the FDR < 0.01 were required for positive protein hits. The label-free quantification (LFQ) method was used to quantify protein. FDR value was also used to correct the *p* value (FDR < 0.05). As LFQ > 2 and *p* ≤ 0.05, the protein was considered to be the differentially expressed proteins (DEPs). FungiFun (Priebe et al., 2011) and KAAS (Kanehisa et al., 2008) were used for GO functional enrichment and KEGG pathway analyses, respectively.

### Reverse Transcription and qRT-PCR Analysis

After treated with the DNase I Kit to remove residual genome DNA, reverse transcription was performed using total RNA and a two-step cDNA synthesis kit (Takara, Dalian, China). Quantitative PCR assays were performed using the Analytic Jena Q-tower system (Analytik-Jena, Jena, Germany) with the 20-μl reaction system, including 5 μl complementary DNA (cDNA) product, 0.5 μl each primer (Supplementary Table S7), and 10 μl SYBR Green mix (Takara, Dalian, China). The PCR program was settled as one cycle of 3 min at 95°C followed by 40 cycles of 10 s at 95°C and 40 s at 65°C, and melting curve analysis was performed from 60 to 90°C with 0.5°C incremental increases. The *18S* rRNA gene was used as the internal reference gene. Quantification of mRNA levels was based on the CT value and calculated with 2^–ΔΔ*CT*^ method.

### Statistical Analysis

All experiments were repeated with three independent biological replicates. The results are presented as the means with standard deviations. One-way analysis of variance (ANOVA) was employed for determining statistical significance of mycelia weight and AFB_1_ production by SPSS 18.0, and Tukey’s test with *p* < 0.05 was used for statistically analyses. Student’s *t* test were applied for comparing the difference of reverse transcription quantitative PCR (RT-qPCR), and differences were assessed by *p* < 0.05.

## Results

### Different Crop Substrates Significantly Affect AFB_1_ Production

**FIGURE 1:**
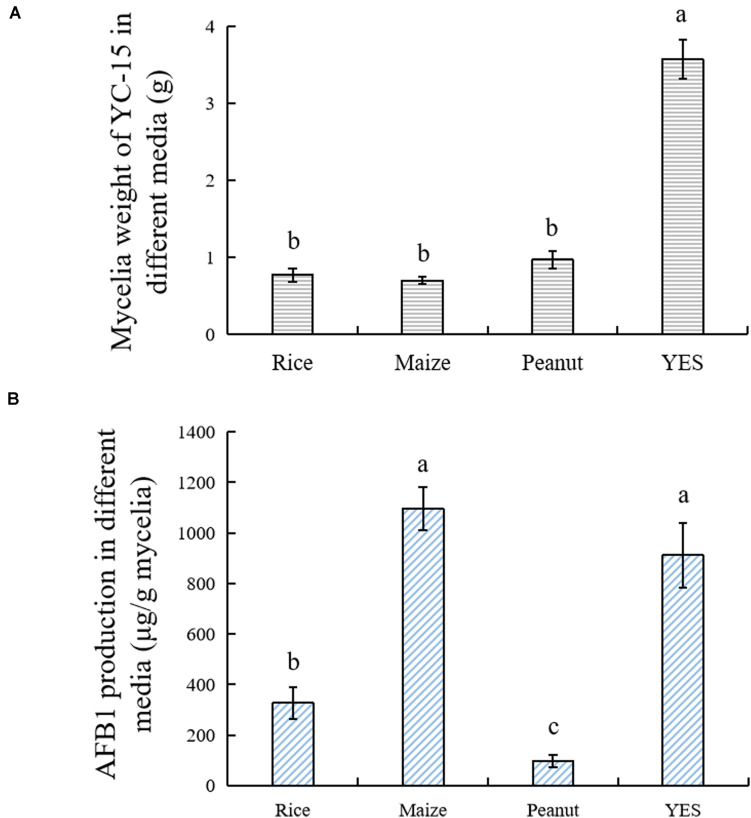
The growth and AFB_1_ production of *A. flavus* YC-15 in different media. The strain was cultivated in four different media with 200 rpm at 28°C. The **(A)** mycelia dry weight and the **(B)** AFB_1_ production were measured after 7 days cultivation. All experiments were performed in three independent replicates. The results were presented as mean ± SD, and error bar indicated SD. The Tukey’s test was used for difference analyses and *p* < 0.05 was considered as statistical difference.

No significant difference in the dry mycelial weights was detected among the three crop media, whereas the mycelial weights in YES medium were clearly higher compared with the crop substrates (Figure 1A). Among three crop substrates, AFB_1_ yields were the highest in the maize broth and lowest in the peanut broth (Figure 1B).

### Transcriptomic Analyses of *A. flavus* in Diverse Media

Three biological replicates were taken for each medium, and a total of 12 libraries were acquired. On average, 50.03, 49.75, 57.89, and 55.99 million raw reads were generated from *A. flavus* in rice, maize, peanut, and YES media, respectively and 47.27, 47.29, 55.46, and 53.71 million clean reads were obtained after quality filtering, respectively (Supplementary Table S1). Furthermore, 92.82, 93.43, 93.41, and 93.31% of clean reads from these four media were mapped to the genome of *A. flavus* NRRL3357, respectively and at least 85.5% of clean reads were mapped to the exon regions. All these data suggest that the RNA-seq data could precisely depict the gene transcription of *A. flavus* strain YC-15 in different substrates.

**FIGURE 2:**
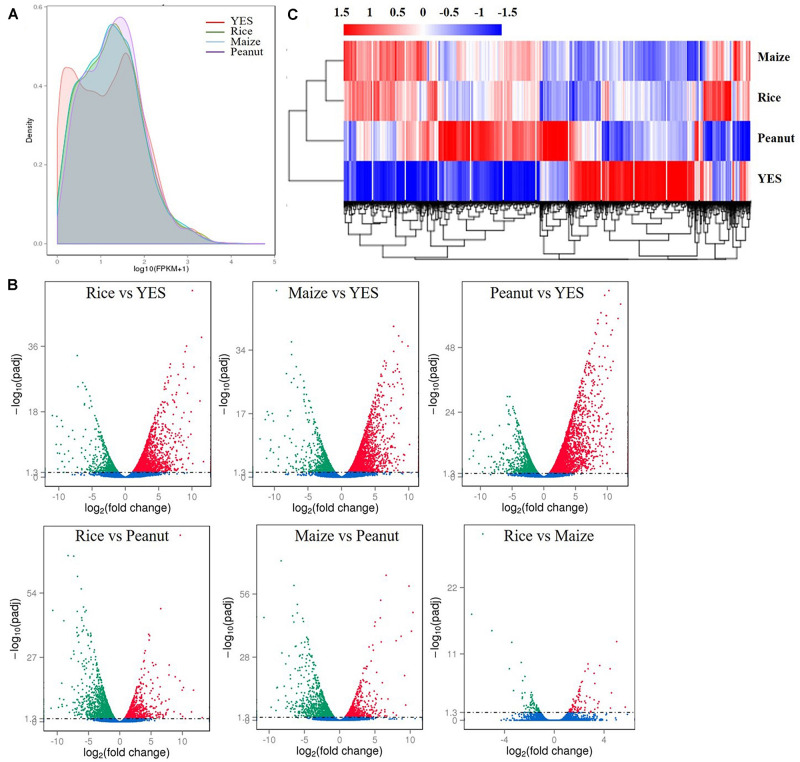
Transcriptome data analysis of *A. flavus* YC-15 in different substrates. **(A)** The fragments per kilobase of transcript per million mapped reads (FPKM) density distribution of *A. flavus* genes in different media. The final FPKM values are the mean values of three independent replicates. Different color curves stands for the diverse crops. **(B)** The volcano plots of the pairwise comparisons of different treatment. The differentially expressed genes (DEGs) in six comparison groups were showed as the red spots (upregulation) and green spots (downregulation), and no significantly changed genes were showed with blue. **(C)** Cluster analysis of DEGs in different media. The values of log_10_ (FPKM + 1) were transformed and clustered. The genes with similar function or in same metabolism pathway, would be clustered into one branch. High expressed genes were in red, and low expressed genes showed with blue.

According to the fragments per kilobase of transcript per million mapped reads (FPKM) values, the density distribution curve of YES medium showed a bimodal curve, which was quite different from the other three groups (Figure 2A). Pairwise comparisons of four media samples were made, and six volcano plots were obtained (Figure 2B). In the rice versus peanut group, the transcription levels of 1,256 genes were upregulated and 1,581 genes were downregulated, whereas those of 1,058 genes were upregulated and 1,332 genes downregulated in the maize versus peanut group. The smallest difference was observed in rice versus maize group with only 173 DEGs including 88 upregulated and 85 downregulated genes. The transcriptional expressions in the three crop substrates were all significantly different from that in YES medium, and thousands of DEGs were identified (Figure 2B). Heat map clustering of the DEGs also showed the obvious differences between YES and crop media. The expression pattern in peanut medium was apparently different from those in rice and maize substrates, whereas it was similar between the rice and maize substrates (Figure 2C).

Kyoto Encyclopedia of Genes and Genomes analysis indicated that the DEGs were enriched in genes involved in biosynthesis of secondary metabolites in the three comparison groups of rice vs. YES, maize vs. YES, and peanut vs. YES, while they were enriched in metabolic pathways and the biosynthesis of secondary metabolites in the rice vs. peanut and maize vs. peanut groups. However, the DEGs were enriched in the biosynthesis of secondary metabolites and arginine and proline metabolism in the rice vs. maize group (Supplementary Figure S1). The GO annotation analysis showed that the DEGs in the comparisons of the crop media vs. YES medium were predominantly enriched in biological process. The DEGs between the different crop substrates were mainly enriched in cellular components, including integral component of membrane, intrinsic component of membrane, membrane part, and membrane (Supplementary Figure S2).

### Intracellular and Extracellular Proteome Analyses of *A. flavus* in Different Media

**FIGURE 3:**
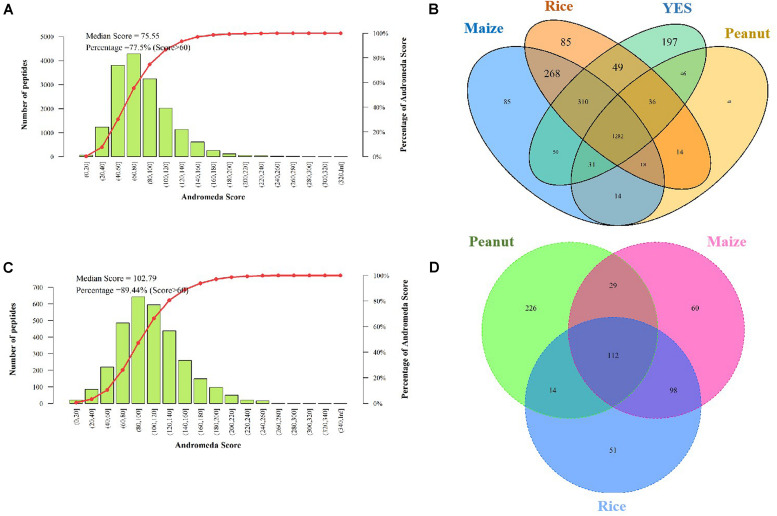
Assessment and identification of proteome data. **(A)** The Andromeda score distribution of intracellular proteome data. The Andromeda score was calculated with Andromeda software to evaluate the availability of MS data. The scores of the major peptides more than 60 means the data are available. **(B)** Venn diagram of the intracellular protein number from four different media. **(C)** The Andromeda score distribution of extracellular proteome data. **(D)** Venn diagram of the extracellular proteins number from three crop substrates.

The Andromeda scores of 77.55 and 89.44% peptides, which were identified in the intracellular and extracellular proteomes, respectively were >60 points (Figures 3A,C), indicating that the MS data were adequate for the subsequent analysis. A total of 2,923 proteins were discovered in the intracellular proteome data, with 2,156, 2,151, 1,681, and 2,005 proteins identified in the rice, maize, peanut, and YES substrates, respectively. Of these, 1,282 shared proteins were identified in the four substrates (Figure 3B). In contrast, only 631 proteins were identified in extracellular proteome, including 275, 299, and 381 proteins in the rice, maize, and peanut substrates, respectively. However, no detectable extracellular proteins were detected in the YES supernatant (Figure 3D).

Both the significantly altered proteins and the consistently present or absent proteins were regarded as DEPs. Among the intracellular proteins, 981, 962, and 1,061 DEPs were identified in the rice vs. YES, maize vs. YES, and peanut vs. YES groups, respectively (Table 1). A total of 1,124 and 1,120 DEPs were identified in the rice vs. peanut and maize vs. peanut groups, respectively. However, the number of DEPs in the maize vs. rice group was only 554. In comparisons of extracellular proteomes, 410 DEPs were identified in the rice vs. peanut group, 409 DEPs were in the maize vs. peanut group, and 177 DEPs were in the maize vs. rice group (Table 1).

**TABLE 1:** The number of differentially expressed proteins (DEPs) in different comparison groups from intracellular and extracellular proteomes analyses.

Comparisons	Significantly changing		Consistent presence/absence difference		Total differential expression proteins (DEPs)
		
	Increased	Decreased	Increased	Decreased	
Rice vs. YES (intracellular)	112	68	476	325	981
Maize vs. YES (intracellular)	98	60	475	329	962
Peanut vs. YES (intracellular)	100	71	283	607	1,061
Rice vs. peanut (intracellular)	92	76	715	241	1,124
Maize vs. Peanut (intracellular)	84	76	715	245	1,120
Maize vs. Rice (intracellular)	101	86	181	186	554
Rice vs. Peanut (extracellular)	3	3	149	255	410
Maize vs. Peanut (extracellular)	4	7	158	240	409
Maize vs. Rice (extracellular)	14	9	89	65	177

**FIGURE 4:**
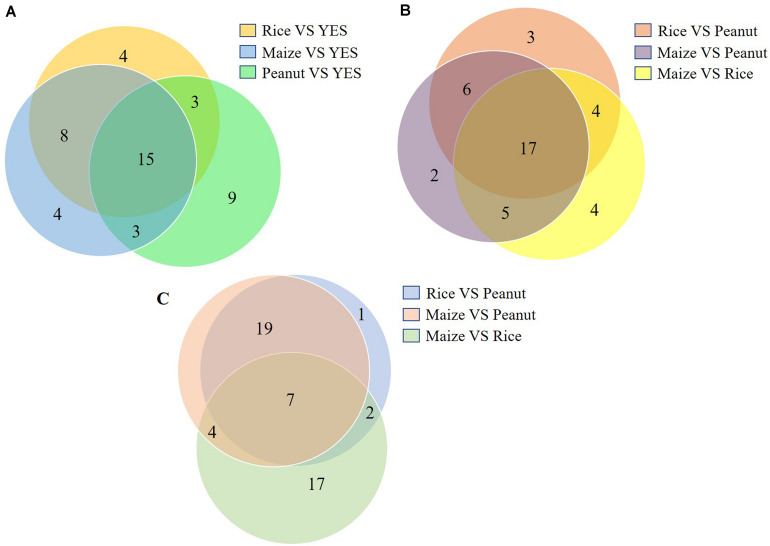
Venn diagram of the number of Kyoto Encyclopedia of Genes and Genomes (KEGG) pathway in differential substrates. **(A)** The number of enriched KEGG pathways in the intracellular-proteome comparisons between YES medium and three crop substrates. **(B)** The number of enriched KEGG pathways in the intracellular-proteome comparisons among three crop substrates. **(C)** The number of enriched KEGG pathways in the extracellular-proteome comparisons among three crop substrates.

The top 20 DEP-enriched KEGG pathways are shown in Supplementary Figures S3, S5. The GO annotation analysis showed that the DEPs involved in metabolic process, cellular process, catalytic activity, binding, cell, and cell part were enriched in all six comparisons of the intracellular proteomes and in three comparisons of the extracellular proteomes (Supplementary Figures S4, S5). As shown in Figure 4, among the top 30 DEP-enriched KEGG pathways, 23 pathways were identical between the rice vs. YES group and maize vs. YES group, and 15 pathways were identical in the rice vs. YES, maize vs. YES, and peanut vs. YES groups (Figure 4A). Twenty-three identical pathways of the intracellular proteome were in the rice vs. peanut and maize vs. peanut groups, while 26 identical pathways of the extracellular proteome were in these two groups (Figures 4B,C), suggesting that the growth and metabolism of *A. flavus* in the crop media were similar, especially in the rice and maize substrates.

### Correlation Analyses of Transcriptomes and Proteomes

**FIGURE 5:**
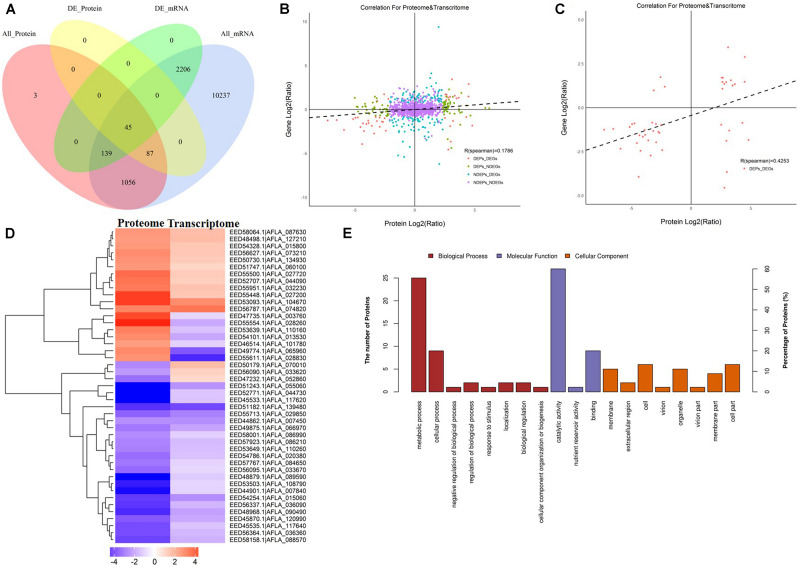
The correlation analyses transcriptomes and proteomes of maize and peanut substrates. **(A)** The Venn diagram of the number of messenger RNAs (mRNAs) and proteins in comparison of maize and peanut substrates. All_Protein, DE_Protein, All_mRNA, and DE_mRNA stand for all quantifiable proteins, significantly different proteins, all quantifiable genes, and significantly different genes, respectively. **(B)** The scatter diagram of the correlation for all proteins and mRNAs. **(C)** The scatter diagram of the correlation for differentially expressed proteins (DEPs) and differentially expressed genes (DEGs). **(D)** Cluster analysis of correlated proteins/genes. Upregulated genes are shown in red, and downregulated genes are in blue. The different shades stand for the different change fold. **(E)** The GO annotation of the 45 correlated proteins/genes.

Since AFB_1_ production was significantly lower in the peanut substrate than in the maize substrate, the transcriptome and proteome correlation analyses in the maize vs. peanut group were examined. Among 1,330 DEPs that were detected in comparison of maize vs. peanut, only 45 proteins and their correspondent mRNA simultaneously showed significant changes (Figure 5A). Low level correlations (*R* = 0.1786) were observed for all the quantified transcripts and proteins (Figure 5B), whereas the correlation index was moderate between the DEGs and DEPs (*R* = 0.4253) (Figure 5C). In a cluster analysis of the 45 correlated DEGs and DEPs (Supplementary Table S2), 35 of the transcripts/proteins showed the same trend of change (12 upregulated and 23 downregulated). However, the other 10 transcripts/proteins presented the change with opposite trend (Figure 5D). Among the 45 DEGs/DEPs, two genes (AFLA_087630 and AFLA_090490) were associated with trehalose metabolism, suggesting that the trehalose metabolism may be relevant with the AFs regulation in different crop substrates.

The KEGG pathway starch and sucrose metabolism, caffeine metabolism, and purine metabolism were enriched among the 45 correlated DEGs and DEPs (data not shown). The GO annotation of these 45 DEGs/DEPs showed that metabolic process (biological process) and catalytic activity (molecular function) were clearly enriched at GO level 2 (Figure 5E). Moreover, GO level 3 enrichment showed that the correlated DEGs/DEPs were significantly enriched in the oxidation–reduction process, nitrogen compound metabolic process, cellular metabolic process, primary metabolic process, and organic substance metabolic process in biological process, and enriched in oxidoreductase activity, transferase activity, and hydrolase activity in molecular function (Supplementary Table S3).

### Analysis of the Expressions of AFs Biosynthetic Cluster (Cluster 54^#^) Genes

The transcriptional levels of almost all AFs synthesis genes were significantly reduced when *A. flavus* was grown on the crop substrates rather than YES medium (Table 2). Pairwise comparisons of the three crop media indicated that 23 and 21 AFs synthesis genes were upregulated in the maize vs. peanut group and the rice vs. peanut group, respectively (Table 2). The result suggests that maize and rice substrates induce the expression of the cluster genes compared to peanut substrate. Among these genes, the expression of *aflA* and *aflB* in maize substrate showed significant increase by 1.96- and 2.94-fold, respectively. The expression of *aflA* and *aflB* were also higher in rice than in peanut substrate (1.72- and 2.66-fold). Furthermore, *aflC*, the PKS-encoding gene of the AFs biosynthesis cluster, showed clearly elevated transcription in the maize and rice substrates. Based on a comparison of LFQ intensity, the AflC protein levels were also 2.66- and 3.26-fold higher in the maize and rice media than the peanut medium, respectively (Supplementary Table S4). However, the regulator genes *aflR* and *aflS* only showed slight upregulation in the maize and rice media compared with the peanut medium. Interestingly, the putative acetyl-CoA carboxylase (ACCase) gene (*accA*, AFLA_046360), involved in malonyl-CoA synthesis, was upregulated at both the transcriptional and translational levels in the maize and rice substrates compared with peanut medium (Table 2 and Supplementary Table S4).

**TABLE 2:** The transcriptional expression changes of aflatoxin synthesis genes in different substrates.

Gene symbol	Gene		log_2_FC	log_2_FC	log_2_FC	log_2_FC	log_2_FC	log_2_FC
(AFLA_xxx)	name	Gene function	(R/Y)	(M/Y)	(P/Y)	(R/P)	(M/P)	(R/M)
139100	*aflYe*	Ser–Thr protein phosphatase family	–1.79	–2.00	–1.26	–0.53	–0.74	0.21
139110	*aflYd*	Sugar regulator	–1.24	–1.17	–1.27	0.03	0.11	–0.08
139120	*aflYc*	Glucosidase	–0.48	–0.08	–0.39	–0.08	0.32	–0.40
139130	*aflYb*	Putative hexose transporter	0.42	0.30	0.17	0.25	0.12	0.12
139140	*aflYa*	NADH oxidase	–0.79	–0.69	–0.80	0.01	0.10	–0.09
139150	*aflY*	Hypothetical protein	–2.51	–2.37	–2.34	–0.16	–0.03	–0.14
139160	*aflX*	Monooxygenase	–2.11	–2.76	–3.59	1.48	0.83	0.65
139170	*aflW*	Monooxygenase	–1.64	–2.16	–2.40	0.76	0.24	0.52
139180	*aflV*	Cytochrome p450 monooxygenase	–3.48	–3.40	–4.20	0.72	0.81	–0.08
139190	*aflK*	VERB synthase	–3.65	–4.36	–4.61	0.47	0.25	0.37
139200	*aflQ*	Cytochrome P450 monooxygenase	1.52	–3.11	–0.74	–2.49	–2.37	0.24
139210	*aflP*	O-methyltransferase A	–1.24	–4.39	–2.40	1.16	–1.99	3.15
139220	*aflO*	O-methyltransferase B	–3.10	–6.01	–1.68	–1.42	–4.33	2.91
139230	*aflI*	Cytochrome P450 monooxygenase	–2.88	–1.62	–2.96	0.08	1.34	–1.26
139240	*aflLa*	Hypothetical protein	–2.66	–3.66	–2.54	–0.12	–1.13	1.01
139250	*aflL*	P450 monooxygenase	–6.33	–6.59	–3.18	–3.15	–3.41	0.26
139260	*aflG*	Cytochrome P450 monooxygenase	–2.27	–2.52	–2.78	0.51	0.26	0.26
139270	*aflNa*	Hypothetical protein	–0.65	–1.01	–1.27	0.63	0.27	0.36
139280	*aflN*	Monooxygenase	–2.23	–2.78	–2.62	0.40	–0.16	0.55
139290	*aflMa*	Hypothetical protein	–3.30	–3.25	–2.48	–0.81	–0.77	–0.04
139300	*aflM*	Ketoreductase	–6.35	–7.72	–4.60	–1.76	–3.12	1.36
139310	*aflE*	NOR reductase	–4.36	–5.09	–3.68	–0.68	–1.40	0.72
139320	*aflJ*	Esterase	–0.65	–1.37	–1.93	1.28	0.56	0.72
139330	*aflH*	Short chain alcohol dehydrogenase	–1.39	–2.08	–2.08	0.69	0.00	0.69
139340	*aflS*	Pathway regulator	–3.27	–3.86	–3.76	0.49	–0.10	0.59
139360	*aflR*	Transcription activator	–1.99	–2.78	–2.39	0.40	–0.38	0.78
139370	*aflB*	Fatty acid synthase beta subunit	–0.49	–0.35	–1.90	1.41	1.56	–0.14
139380	*aflA*	Fatty acid synthase alpha subunit	0.89	1.08	0.11	0.78	0.97	–0.19
139390	*aflD*	Reductase	–1.51	–1.32	–2.11	0.60	0.80	–0.19
139400	*aflCa*	Hypothetical protein	–0.49	0.50	–0.42	–0.07	0.92	–0.99
139410	*aflC*	Polyketide synthase	1.87	2.05	0.70	1.17	1.35	–0.18
139420	*aflT*	Transmembrane protein	–0.96	–1.38	–1.60	0.64	0.22	0.42
139430	*aflU*	P450 monooxygenase	–2.44	–2.10	–2.69	0.25	0.59	–0.34
139440	*aflF*	Dehydrogenase	–1.82	–1.54	–2.24	0.42	0.70	–0.28
046360	*accA*	Acetyl-CoA carboxylase putative	–1.08	–1.14	–2.35	1.27	1.21	0.06

*R, M, P, and Y stand for rice, maize, peanut, and YES media, respectively.*

**FIGURE 6:**
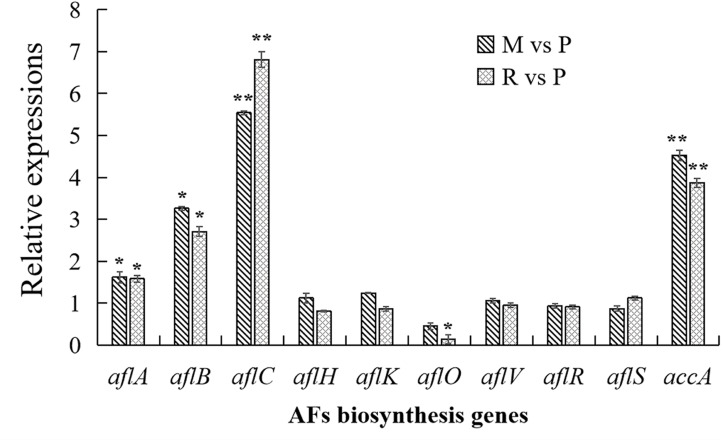
Transcriptional expressions of aflatoxins (AFs) biosynthesis genes with quantitative reverse transcription PCR (qRT-PCR) analysis. The transcriptional expressions of AFs genes in different crop media were detected by qRT-PCT analysis. The transcripts from maize and rice media were compared with that from peanut substrate, showing as M vs. P and R vs. P. Three independent experiments were performed in each substrate, and data were presented as means ± SD. *t* tests were applied for comparing the difference with *p < 0.05 and **p < 0.01.

In order to confirm the result of AFs cluster transcripts, RT-qPCR analyses were performed in this study (Figure 6). Similar with transcriptome data, *aflC* expression showed the most strongly improved in maize and rice substrates than in peanut. The expressions of *aflB*, *aflC*, as well as *accA* were also significantly upregulated in maize and rice compared with that in peanut. Other detected genes were not significantly different in diverse substrates, with the exception that *aflO* expression showed significantly decreased in rice medium than in peanut medium.

### Carbon-Metabolism-Related Genes Are Differentially Expressed in Maize and Peanut Media

Compared with the peanut medium, the levels of both AflC and ACC were upregulated in the maize medium, suggesting that the acetyl-CoA and malonyl-CoA levels may contribute to the difference in AFs production in the different substrates. The acetyl-CoA-related DEGs were also enriched in fatty acid degradation (afv00071), glycolysis (afv00010), and citrate/tricarboxylic acid (TCA) cycle (afv00020) (Supplementary Table S5). AFLA_031570 and AFLA_035290, encoding the core proteins of the pyruvate dehydrogenase system, were significantly upregulated in the maize substrate than in the peanut substrate, implying that more pyruvate was decomposed into acetyl-CoA in the maize medium (Supplementary Table S5). AFLA_049290 and AFLA_107660, the genes in afv00020, were downregulated in the maize substrate, suggesting that less acetyl-CoA was consumed in the TCA cycle than in the peanut medium (Supplementary Table S5). In the fatty acid degradation process (afv00071), the transcriptional levels of the P450 family fatty acid hydroxylase (AFLA_085490) and acyl-CoA oxidase (AFLA_115890) were upregulated in the maize medium, but the acyl-CoA dehydrogenase family protein (AFLA_049020) and reduced nicotinamide adenine dinucleotide phosphate (NADPH) flavin oxidoreductase (AFLA_077220) were downregulated (Supplementary Table S5).

We also noted that 23 DEGs were enriched in the starch pathway and sucrose metabolism (AFV00500). AFLA_023490 (α-amylase gene), AFLA_026140 (α-amylase gene), and AFLA_081340 (glycogen debranching enzyme gene) were significantly increased in the maize substrate (Supplementary Table S5). Pyruvate dehydrogenase (AFLA_035290) and 6-phosphogluconate dehydrogenase (AFLA_128510), the key enzymes of pyruvate metabolism (AFV00620) and the pentose phosphate pathway (AFV00030), respectively were both upregulated in the maize medium (Supplementary Table S5).

### Varying Expression of Global Regulators of AFs Production in Different Substrates

Expressions of the main global regulators associated with AFs production in the maize and peanut substrates are listed in Supplementary Table S6. As the central protein of the velvet complex (VelB/VeA/LaeA), VeA transcription was significantly higher in the maize medium than in the peanut medium, whereas the expressions of neither *velB* nor *laeA* differed significantly. The oxidative stress-related transcription factors AtfA, AtfB, AP-1, and MsnA were similar in the different substrates. Moreover, the transcripts of the MAPK pathway genes also showed similar expression except *bck1*, which encodes MAPK kinase and was significantly elevated in the maize substrate. Expressions of all the oxylipin genes *ppoA*, *ppoB*, and *ppo*C were lower in the maize substrate than in the peanut substrate, of which the *ppoB* expression were significantly reduced. It is remarkable that 7 of the 19 GPCR signal transduction system genes showed significantly differential expressions. Of these, *AfPXG*, *gprG*, and *gprJ* were upregulated and *gprC*, *gprH*, *gprM*, and *gprR* were downregulated in the maize substrate. The regulators involved in carbon source (CreA), nitrogen signal (AreA), pH (PacC), and the cAMP signal (PkaR, PkaC, CapA, SomA, and Sok1) showed no significant transcriptional changes between the maize and peanut media. However, *snf1*, which is involved in CreA phosphorylation, was significantly upregulated in the maize substrate, implying that posttranslational modification may play an important role in AFs regulation.

## Discussion

*Aspergillus flavus* is a saprophytic fungus that can invade almost all foods and feeds during the pre- and post-harvest. It can produce the most toxic and carcinogenic naturally occurring compounds, AFs (Amaike and Keller, 2011). To mimic the production of AFs in different crops, maize, rice, and peanuts were used as the substrates for the *A. flavus* cultivation. The significant differences in AFB_1_ levels were observed in the three crop media, while the fungal growth was similar (Figure 1). Higher AFB_1_ levels were detected in maize and rice media, but AFB_1_ yield in peanut substrate showed significantly decreased (Figure 1B). Our results were in agreement with the previous investigations. Crops detection showed that AFB_1_ contamination was more severe in maize than in peanut (Njumbe et al., 2014). By artificial inoculation, AFs production was also higher in cereals (wheat, maize, and rice) than in nuts (almond, walnut, and peanut) (Iqbal et al., 2006).

Based on the transcriptome and proteome data, the similar growth and metabolism were observed in maize vs. rice group, but obvious differences were noticed in maize vs. peanut group. We supposed that the differential expressions of *A. flavus* genes and proteins in different crop substrates were caused by the different nutrition compositions. The main nutrient compositions of maize and rice are similar, and both include starch, proteins, fatty matter, total sugars, etc. Of them, the starch accounts for more than 70% of dry weight of maize and rice (Kouakou et al., 2008). In contrast, the nutrients in peanut are markedly different from maize and rice and include 20–30% crude proteins, ∼50% lipids, and ∼38% total carbohydrates containing only 12.5% starch (Toomer, 2018). Therefore, the different compositions of maize, rice, and peanut may contribute to the differential expression of genes and proteins, and the contents of carbohydrates may be relevant to the AFs production in diverse crop substrates.

For AFs cluster, 21 genes were up regulated at the transcription level in the maize substrate compared with peanut substrate, leading to the higher AFB_1_ level in the maize substrate (Table 2). Fatty acid synthase is responsible for the synthesis of the AFs polyketide backbone (Mahanti et al., 1996), and the formation of the hexanoyl unit from acetyl-CoA and malonyl-CoA is the first step of AFs biosynthesis (Minto and Townsend, 1997). The *aflB*, which encodes the fatty acid synthase beta subunit, was strongly upregulated. The *aflA*, the fatty acid synthase alpha subunit gene, also displayed increased mRNA levels. As the polyketide synthase of AFs biosynthesis cascade, AflC (PksA) is required for the biosynthesis of the first stable intermediate, norsolorinic acid (NOR) (Yu et al., 2004). Thus, *aflC* is regarded as one of the most important structural gene in the AFs biosynthesis cluster (Papa, 1982; Yu et al., 2004). In the maize medium, *aflC* was significantly induced at both the transcriptional and translational levels (Table 2 and Supplementary Table S4). Similarly, *aflA*, *aflB*, and *aflC* were also upregulated in rice medium (Table 2 and Figure 6). These results suggest that the expression changes in the initial steps genes, especially *aflC*, may contribute to the increase in AFs production in different crop media. However, the expression of the two key cluster regulator genes *aflR* and *aflS* showed no significant changes in the various crop media, implying that changes in *aflA*, *aflB*, and *aflC* expression are not caused by the change in *aflR* and *aflS* (Table 2 and Figure 6), and might be induced by the levels of AFs precursors (acetyl-CoA and malonyl-CoA). Furthermore, the expression of *accA* (AFLA_046360), which is responsible for malonyl-CoA synthesis from acetyl-CoA (Morrice et al., 1998), was clearly upregulated in both the maize and rice media (Table 2 and Figure 6). The overexpression of ACCase in *A. terreus* also increased both malonyl-CoA and the polyketide secondary metabolite, lovastatin (Hasan et al., 2018). These results confirmed that the increase in AFs production in maize and rice were correlated closely with the upregulation of genes involved in the early steps of AFs biosynthesis and the higher levels of the precursors.

Acetyl-CoA is the key element required for the synthesis of polyketide chemicals, and its levels positively correlate with the AFs yield (Vahlensieck et al., 1994; Narasaiah et al., 2006; Hasan et al., 2018). In *A. flavus*, fatty acid β-oxidation (AFV00071) and sugar glycolysis (AFV00010) are the vitally contributory pathways for acetyl-CoA biosynthesis (Zhao et al., 2018). In this study, the transcriptional expressions of pyruvate decarboxylase gene (AFLA_031570) and pyruvate dehydrogenase gene (AFLA_035290), which are responsible for the transformation from pyruvate to acetyl-CoA, were significantly increased in maize substrate. The key genes of pyruvate metabolism and the pentose phosphate pathway were also upregulated, whereas several genes of the TCA cycle were downregulated (Supplementary Table S5). In summary, compared with the peanut substrate, the major genes involved in carbon source decomposition and acetyl-CoA synthesis were upregulated in the maize medium, and the main genes involved in acetyl-CoA catabolism were decreased, leading to the accumulation of acetyl-CoA.

Compared with the peanut substrate, the maize substrate is rich in starch (Kouakou et al., 2008; Toomer, 2018). The starch hydrolysis provides the basic carbon source for the growth of *A. flavus* and its secondary metabolite synthesis. Among the numerous hydrolases, α-amylase is the key enzyme responsible for the hydrolysis of α-linked polysaccharides like starch (Gupta et al., 2008). A previous study has shown that after the addition of extra α-amylase, the AFs production in the maize substrate was significantly improved (Vidal et al., 2018). The suppression of the α-amylase gene *amy1* in *A. flavus* effectively reduced AFs contamination (Gilbert et al., 2018). In the present study, the expression of α-amylase (AFLA_023490 and AFLA_026140) (Supplementary Table S5) were significantly upregulated in maize vs. peanut group, and the α-amylase protein (AFLA_026140) was also significant increased both at intracellular and extracellular proteome (data not shown). The higher Amy1 level in maize substrate may contribute to the improvement of AFB_1_ production. In addition, the previous study showed that alpha-amylase was positively regulated by LaeA (Lv Y. et al., 2018), but we did not find significant difference of *laeA* transcript, suggesting that hydrolase expression in different substrates may be modulated with the other regulating way.

The correlation analysis between transcriptomic and proteomic of the *A. flavus* in maize and peanut substrates was performed. A low correlation of transcriptome and proteome data was obtained in this study (Figures 5B,C), and only 45 DEGs and their corresponding DEPs were observed (Figure 5A). The similar low correlations have been reported by Barker et al. (2012) and Bai et al. (2015) in *Aspergillus fumigatus* and *A. flavus*, they thought that the posttranscriptional modification might play a critical role in the regulation of the protein level, and the mRNA changes provided only limited contribution to the protein changes. The insufficient number of proteins also contributed to the low correlation. Although proteomic analysis can provide straightforward message about the protein expression and metabolism change, this technology suffers from its inherent shortcomings, such as extraction losses, protein dissolution, fractionation losses, etc. (Resch et al., 2006). In this study, the 2,390 DEGs were detected from 13,770 transcripts, but <10% of 1,330 detected proteins were identified as DEPs (132) (Figure 5A). The third possible reason is that the synthesis and turnover rate of proteins and mRNAs can differ in various cell stages. In *Saccharomyces cerevisiae*, Rossignol et al. (2009) reported that the selective translation of the mRNAs was noticed as the cells entered into stationary phase, which led to the insufficient correlation. In this study, many DEGs could not lead to its protein differential expression, especially 10 of the transcripts showed the opposite expression patterns to their cognate DEPs (Supplementary Table S2). Taken together, the limited number of proteins, the stabilization of mRNA and protein, and the posttranscriptional or posttranslational modification could be contributed to the low correlation between the transcriptome and proteome data.

We also attempted to find some information from the 45 correlated DEGs/DEPs. Eight genes involved in carbon metabolism and two genes involved in trehalose metabolism were identified among the 45 DEGs/DEPs (Figure 5D), suggesting again that the different AFs production in diverse crop substrates may be caused by the carbon metabolism changes. The *aldA* (AFLA_108790), encoding the aldehyde dehydrogenase, was noticed among the 45 genes. After treatment with ethanol, the *aldA* transcription increased and the AFs production was suppressed in *A. flavus*. Thus, the *aldA* expression is negatively associated with AFs production (Ren et al., 2020). In the present study, both the mRNA and protein level of AldA were significantly reduced in maize substrate (Supplementary Table S2). The results suggest that the ethanol metabolism of *A. flavus* may be suppressed in maize substrate. In addition, the dimethylallyl tryptophan synthase gene (AFLA_139480) was also downregulated in maize substrate (Supplementary Table S2). It is interesting to note that its physical locus is adjacent to the downstream of the AF biosynthetic cluster.

Many physiological and genetic studies have provided strong evidence that oxidative stress could promote the AFs production (Reverberi et al., 2005; Lv C. et al., 2018; Wang et al., 2019). The oxidative stress-related transcription factors (TFs) AtfB, SrrA, AP-1, and MsnA physically bind to the promoter regions of the oxidative stress response genes, as well as the AFs biosynthetic genes, and participate in the activation of the AFs cluster together with AflR (Hong and Wee, 2013). However, in the present study, no significant changes were identified in the oxidative-stress-related TFs or oxidative-stress-response genes (Supplementary Table S6). The result suggests that no oxidative disturbance was triggered by the different substrates and that the changes in AFs synthesis genes and AFs production were independent of these TFs. VeA combined with VelB and LaeA to form the velvet complex, which responds to light signals and controls fungal development and secondary metabolism (Bayram et al., 2008). The deletion of *veA* and *laeA* caused an obvious decline in AFs (Amaike and Keller, 2009), and the suppression of AFs biosynthesis by cinnamaldehyde and eugenol was also relied on VeA and the velvet complex (Lv Y. et al., 2018; Wang et al., 2019). Our results showed that the transcription of *veA* was significantly increased in the maize medium, confirming VeA is a positive regulator of AFs production. However, the expression of the other velvet regulator genes, *velB* and *laeA*, did not differ among the different substrates in this study.

The MAPK pathway is a critical mechanism for fungal environmental adaptation by protein phosphorylation. In *A. flavus*, several environmental conditions induce AFs biosynthesis by regulating the MAPK cascade system (Ren et al., 2016). The AFB_1_ level in the Δ*ste11* mutant were significantly reduced (Ren et al., 2016). However, there were no transcription differences for the MAPK genes in the maize and peanut substrate (Supplementary Table S6). In addition, the mRNA level of *amy1* was significantly unregulated in the maize substrate, whereas CreA, the transcriptional regulator of *amy1*, showed no obvious difference at either the transcriptional or translational level (Supplementary Table S6). However, protein kinase Snf1, regulating CreA phosphorylation and its intracellular localization (Adnan et al., 2017), was significantly increased in the maize medium (Supplementary Table S6). It is logical to postulate that the differential expression of *snf1* affected the phosphorylation level of CreA, activated its function, and regulated the *amy1* transcription on diverse substrates. With the exception of phosphorylation, lysine acetylation is also a critical posttranslational modification and can directly regulates AFs biosynthesis through the lysine acetylation of AflA and AflB (Lv, 2017). All these posttranslational modifications are involved in the regulation of AFs production but independent on the transcriptional and translational expression change.

Membrane-associated proteins regulate several cell functions, including the maintenance of cell shape, extra- and intracellular signal transduction, nutrient and metabolite transportation, and sensing and adaptation to environmental changes. AfPXG, the caleosin in *A. flavus*, has peroxygenase activity and mediates fungal development and AFs accumulation (Hanano et al., 2015). An *AfPXG*-deficient strain showed severely reduced mycelial growth and obviously reduced AFs production (Hanano et al., 2018). Similarly, the result of this study indicated that *AfPXG* expression was significantly higher in AFs-inducing medium (maize), confirming that AfPXG is a critical positive regulator of AFs biosynthesis. Like oxylipins, the GPCRs also participate in extracellular signal transduction and regulate the expression of downstream genes to allow environmental adaptation (Li et al., 2007). The deletion of *gprK* increased AFs biosynthesis and overexpressed *gprK* reduced AFs production (Caceres et al., 2017). Moreover, when AFs production was suppressed by eugenol and cinnamaldehyde, the GPCR genes *gprC*, *gprF*, *gprK*, *gprM*, and *gprS* were significantly upregulated (Lv C. et al., 2018; Wang et al., 2019). In the present study, 5 of 14 GPCR genes (*gprC*, *gprH*, *gprJ*, *gprM*, and *gprR*) were significantly downregulated in the maize medium (Supplementary Table S6), suggesting that the GPCRs can sense different conditions and are closely related to AFs biosynthesis. Affeldt et al. (2014) reported that the GPCR protein could regulate the AFs biosynthesis by the cAMP-PKA pathway. Taken together, we speculate that membrane proteins could sense the diverse nutrition, transmit the regulatory signal, regulate the downstream gene expressions, and control both the nutrition utilization and the AFs production.

## Conclusion

In this study, *A. flavus* YC-15 were incubated in maize, rice, peanut, and YES media to reveal the mechanisms underlying the changes in AFs production on different crop substrates using transcriptomic and proteomic analyses. Based on previous papers and our results, we conclude that (1) *A. flavus* produced more AFB_1_ in maize/rice substrate than in peanut substrate; (2) the expression of genes involved in the initial steps of AFs biosynthesis was significantly increased in maize substrate; (3) the genes involved in acetyl-CoA accumulation were upregulated, and the genes involved in acetyl-CoA consumption were downregulated, which increased the acetyl-CoA level in maize substrate than in peanut substrate; (4) *veA*, GPCR genes, the trehalose metabolism genes, the aldehyde dehydrogenase gene, and the tryptophan synthase gene may play important roles in the regulation of AFs production in different crop substrates; (5) posttranslational modifications, such as phosphorylation, acetylation, and ubiquitination, may be also involved in the regulation of AFs biosynthesis in diverse crop substrates.

## Data Availability Statement

The datasets generated for this study can be found in the NCBI under accession number PRJNA605915.

## Author Contributions

FX conceived and designed the experiments. XL, YJ, XM, JS, and LM performed the experiments. YL and FX analyzed the data. XL, KM, and FX wrote the manuscript. All authors contributed to the article and approved the submitted version.

## Conflict of Interest

The authors declare that the research was conducted in the absence of any commercial or financial relationships that could be construed as a potential conflict of interest.

## Footnotes

1 https://maxquant.org/

2 https://www.uniprot.org/
