# Supplementary material for: Comprehensive Transcriptome and Proteome Analyses Reveal the Modulation of Aflatoxin Production by Aspergillus flavus on Different Crop Substrates
Source: Front Microbiol. 2020 Jul 14;11:1497. doi: 10.3389/fmicb.2020.01497 (PMC7371938; doi:10.3389/fmicb.2020.01497)
Supplement: Supplementary file 1 [file Data_Sheet_1.docx]

Table S1 The results of assessment and treatment with transcriptome data

| Sample name | Raw reads | Clean reads | Clean bases | Error rate (%) | Q20 (%) | GC content (%) | Total mapped | Total mapped (%) | Multiple mapped | Uniquely mapped |
| --- | --- | --- | --- | --- | --- | --- | --- | --- | --- | --- |
| Rice-1 | 52305350 | 49528454 | 7.43 G | 0.02 | 96.82 | 52.62 | 45832364 | 92.54% | 0.35% | 92.18% |
| Rice-2 | 48426558 | 45915898 | 6.89 G | 0.02 | 97.15 | 53.01 | 42719951 | 93.04% | 0.35% | 92.69% |
| Rice-3 | 49383716 | 46355674 | 6.95 G | 0.02 | 97.10 | 52.74 | 43156292 | 93.10% | 0.34% | 92.76% |
| Maize-1 | 44224312 | 42342330 | 6.35 G | 0.02 | 97.10 | 52.52 | 39545174 | 93.39% | 0.26% | 93.13% |
| Maize-2 | 48072722 | 45076176 | 6.76 G | 0.02 | 97.24 | 53.24 | 42055499 | 93.39% | 0.28% | 93.02% |
| Maize-3 | 56967956 | 54458152 | 8.17 G | 0.02 | 97.12 | 52.37 | 50972576 | 93.60% | 0.27% | 93.33% |
| Peanut-1 | 45339812 | 43068722 | 6.46 G | 0.02 | 97.25 | 52.87 | 40351219 | 93.69% | 0.28% | 93.41% |
| Peanut-2 | 62983806 | 60403770 | 9.06 G | 0.01 | 97.50 | 52.67 | 56102606 | 93.69% | 0.28% | 92.58% |
| Peanut-3 | 65355804 | 62905306 | 9.44 G | 0.01 | 97.52 | 52.70 | 58920656 | 93.67% | 0.27% | 93.40% |
| YES-1 | 52090410 | 50057250 | 7.51 G | 0.02 | 97.19 | 52.75 | 46816385 | 93.53% | 0.35% | 93.17% |
| YES-2 | 67496242 | 64763392 | 9.71 G | 0.01 | 97.64 | 53.24 | 60837113 | 93.94% | 0.46% | 93.48% |
| YES-3 | 48388132 | 46338718 | 6.95 G | 0.02 | 97.07 | 53.00 | 42841425 | 92.45% | 0.44% | 92.01% |

Table S2 The correlated DEGs and DEPs in comparison of maize substrate and peanut substrate

| Protein/mRNA | mRNA change (M/P) | protein change (M/P) | Annotation | KEGG map_ID |
| --- | --- | --- | --- | --- |
| EED58064.1\|AFLA_087630 | up | up | Alpha,alpha-trehalose-phosphate synthase subunit putative | ko00500 |
| EED48498.1\|AFLA_127210 | up | up | pathogenesis associated protein Cap putative |  |
| EED54328.1\|AFLA_015800 | up | up | DUF636 domain protein |  |
| EED56627.1\|AFLA_073210 | up | up | oxalate decarboxylase putative |  |
| EED50730.1\|AFLA_134930 | up | up | mitochondrial phosphate transporter Pic2 putative |  |
| EED51747.1\|AFLA_060100 | up | up | hypothetical protein |  |
| EED55500.1\|AFLA_027720 | up | up | phosphatidyl synthase |  |
| EED52707.1\|AFLA_044090 | up | up | urate oxydase UaZ | ko00365, ko00232 |
| EED55951.1\|AFLA_032230 | up | up | meiotic sister chromatid recombination protein Ish1/Msc1 putative |  |
| EED55448.1\|AFLA_027200 | up | up | xanthine dehydrogenase HxA putative | ko00232,ko00232,ko04146,ko00983 |
| EED53093.1\|AFLA_104670 | up | up | serine peptidase putative |  |
| EED56787.1\|AFLA_074820 | up | up | conserved hypothetical protein |  |
| EED47735.1\|AFLA_003760 | up | down | NACHT domain protein |  |
| EED55554.1\|AFLA_028260 | up | down | exo-beta-1,3-glucanase (Exg1) putative | ko00500 |
| EED53639.1\|AFLA_110160 | up | down | extracellular dipeptidyl-peptidase Dpp4 | ko04974 |
| EED54101.1\|AFLA_013530 | up | down | conserved hypothetical protein |  |
| EED46514.1\|AFLA_101780 | up | down | NADH-cytochrome B5 reductase putative | ko00920 |
| EED49774.1\|AFLA_065960 | up | down | fucose-specific lectin FleA |  |
| EED55611.1\|AFLA_028830 | up | down | FG-GAP repeat protein putative |  |
| EED50179.1\|AFLA_070010 | down | up | conserved hypothetical protein |  |
| EED56090.1\|AFLA_033620 | down | up | RNA binding protein putative |  |
| EED47232.1\|AFLA_052860 | down | up | chaperone/heat shock protein Hsp12 putative |  |
| EED51243.1\|AFLA_055060 | down | down | NAD-dependent formate dehydrogenase AciA/Fdh | ko00680, ko00630 |
| EED52771.1\|AFLA_044730 | down | down | oxidoreductase 2-nitropropane dioxygenase family putative |  |
| EED45533.1\|AFLA_117620 | down | down | conserved hypothetical protein |  |
| EED51182.1\|AFLA_139480 | down | down | dimethylallyl tryptophan synthase putative |  |
| EED55713.1\|AFLA_029850 | down | down | cysteine-rich secreted protein |  |
| EED44862.1\|AFLA_007450 | down | down | O-methyltransferase putative |  |
| EED49875.1\|AFLA_066970 | down | down | conserved hypothetical protein |  |
| EED58001.1\|AFLA_086990 | down | down | cell wall protein putative |  |
| EED57923.1\|AFLA_086210 | down | down | epoxide hydrolase putative |  |
| EED53649.1\|AFLA_110260 | down | down | flavin-binding monooxygenase-like protein |  |
| EED54786.1\|AFLA_020380 | down | down | stomatin family protein |  |
| EED57767.1\|AFLA_084650 | down | down | palmitoyltransferase with autoacylation activity Pfa4 putative |  |
| EED56095.1\|AFLA_033670 | down | down | lipid transfer protein putative |  |
| EED48879.1\|AFLA_089590 | down | down | conserved hypothetical protein |  |
| EED53503.1\|AFLA_108790 | down | down | aldehyde dehydrogenase AldA putative | ko00010, ko00981, ko00280, ko00310, ko00620, ko00071, ko00561 |
| EED44901.1\|AFLA_007840 | down | down | cysteine desulfurylase putative |  |
| EED54254.1\|AFLA_015060 | down | down | phytanoyl-CoA dioxygenase family protein |  |
| EED56337.1\|AFLA_036090 | down | down | homogentisate 1,2-dioxygenase (HmgA) putative | ko00643, ko00350 |
| EED48968.1\|AFLA_090490 | down | down | alpha,alpha-trehalose glucohydrolase TreA/Ath1 |  |
| EED45870.1\|AFLA_120990 | down | down | O-methyltransferase putative |  |
| EED45535.1\|AFLA_117640 | down | down | CipC-like antibiotic stress responsive protein |  |
| EED56364.1\|AFLA_036360 | down | down | mitochondrial dicarboxylate carrier protein putative |  |
| EED58158.1\|AFLA_088570 | down | down | conserved hypothetical protein |  |

M and P stand for maize and peanut media, respectively.

Table S3 Level 3 GO enrichment of DEGs in comparison of maize substrate versus peanut substrate

| Level | GO ID | Term | Type | Sequence number |
| --- | --- | --- | --- | --- |
| 3 | GO:0055114 | oxidation-reduction process | Biological Process | 12 |
| 3 | GO:0006807 | nitrogen compound metabolic process | Biological Process | 8 |
| 3 | GO:0009056 | catabolic process | Biological Process | 4 |
| 3 | GO:0009058 | biosynthetic process | Biological Process | 2 |
| 3 | GO:0019222 | regulation of metabolic process | Biological Process | 1 |
| 3 | GO:0032259 | methylation | Biological Process | 2 |
| 3 | GO:0044237 | cellular metabolic process | Biological Process | 7 |
| 3 | GO:0044238 | primary metabolic process | Biological Process | 9 |
| 3 | GO:0044281 | small molecule metabolic process | Biological Process | 4 |
| 3 | GO:0071704 | organic substance metabolic process | Biological Process | 12 |
| 3 | GO:0007049 | cell cycle | Biological Process | 1 |
| 3 | GO:0016043 | cellular component organization | Biological Process | 1 |
| 3 | GO:0022402 | cell cycle process | Biological Process | 1 |
| 3 | GO:0048523 | negative regulation of cellular process | Biological Process | 1 |
| 3 | GO:0050794 | regulation of cellular process | Biological Process | 2 |
| 3 | GO:0071554 | cell wall organization or biogenesis | Biological Process | 1 |
| 3 | GO:0006950 | response to stress | Biological Process | 1 |
| 3 | GO:0051234 | establishment of localization | Biological Process | 2 |
| 3 | GO:0016491 | oxidoreductase activity | Molecular Function | 12 |
| 3 | GO:0016740 | transferase activity | Molecular Function | 7 |
| 3 | GO:0016787 | hydrolase activity | Molecular Function | 8 |
| 3 | GO:0016829 | lyase activity | Molecular Function | 2 |
| 3 | GO:0016853 | isomerase activity | Molecular Function | 1 |
| 3 | GO:0140096 | catalytic activity, acting on a protein | Molecular Function | 3 |
| 3 | GO:0005515 | protein binding | Molecular Function | 1 |
| 3 | GO:0030246 | carbohydrate binding | Molecular Function | 2 |
| 3 | GO:0008289 | lipid binding | Molecular Function | 1 |
| 3 | GO:0036094 | small molecule binding | Molecular Function | 5 |
| 3 | GO:0043167 | ion binding | Molecular Function | 5 |
| 3 | GO:0048037 | cofactor binding | Molecular Function | 3 |
| 3 | GO:0051540 | metal cluster binding | Molecular Function | 1 |
| 3 | GO:0097159 | organic cyclic compound binding | Molecular Function | 5 |
| 3 | GO:0097367 | carbohydrate derivative binding | Molecular Function | 1 |
| 3 | GO:1901363 | heterocyclic compound binding | Molecular Function | 4 |
| 3 | GO:0043227 | membrane-bounded organelle | Cellular Component | 5 |
| 3 | GO:0043229 | intracellular organelle | Cellular Component | 5 |
| 3 | GO:0036338 | viral membrane | Cellular Component | 1 |
| 3 | GO:0031224 | intrinsic component of membrane | Cellular Component | 4 |
| 3 | GO:0005622 | intracellular | Cellular Component | 6 |
| 3 | GO:0012505 | endomembrane system | Cellular Component | 2 |
| 3 | GO:0044424 | intracellular part | Cellular Component | 5 |

Table S4 Protein changes of the AF biosynthesis cluster genes in different crop substrates

| Gene symbol  (AFLA_xxx) | Gene | Gene function | R/Y  (LFQ) | M/Y  (LFQ) | P/Y  (LFQ) | R/P  (LFQ) | M/P  (LFQ) | R/M  (LFQ) |
| --- | --- | --- | --- | --- | --- | --- | --- | --- |
| 139100 | *aflYe* | Ser-Thr protein phosphatase family protein | \ | \ | \ | \ | \ | \ |
| 139110 | *aflYd* | sugar regulator | \ | \ | \ | \ | \ | \ |
| 139120 | *aflYc* | glucosidase | \ | \ | \ | \ | \ | \ |
| 139130 | *aflYb* | putative hexose transporter | \ | \ | \ | \ | \ | \ |
| 139140 | *aflYa* | NADH oxidase | \ | \ | \ | \ | \ | \ |
| 139150 | *aflY* | hypothetical protein | \ | \ | \ | \ | \ | \ |
| 139160 | *aflX* | monooxygenase | \ | 0.32 | \ | \ | \ | \ |
| 139170 | *aflW* | monooxygenase | \ | \ | \ | \ | \ | \ |
| 139180 | *aflV* | cytochrome P450 monooxygenase | \ | \ | \ | \ | \ | \ |
| 139190 | *aflK* | VERB synthase | 0.38 | 1.70 | 0.28 | 1.37 | 6.15 | 0.22 |
| 139200 | *aflQ* | cytochrome P450 monooxigenase | \ | \ | \ | \ | \ | \ |
| 139210 | *aflP* | O-methyltransferase A | \ | \ | \ | \ | \ | \ |
| 139220 | *aflO* | O-methyltransferase B | \ | \ | \ | \ | \ | \ |
| 139230 | *aflI* | cytochrome P450 monooxigenase | \ | \ | \ | \ | \ | \ |
| 139240 | *aflLa* | hypothetical protein | \ | \ | \ | \ | \ | \ |
| 139250 | *aflL* | P450 monooxygenase | \ | \ | \ | \ | \ | \ |
| 139260 | *aflG* | cytochrome P450 monooxygenase | \ | \ | \ | \ | \ | \ |
| 139270 | *aflNa* | hypothetical protein | \ | \ | \ | \ | \ | \ |
| 139280 | *aflN* | monooxygenase | \ | \ | \ | \ | \ | \ |
| 139290 | *aflMa* | hypothetical protein | \ | \ | \ | \ | \ | \ |
| 139300 | *aflM* | ketoreductase | 0.05 | 0.11 | 0.08 | 0.65 | 1.38 | 0.48 |
| 139310 | *aflE* | NOR reductase | \ | \ | \ | \ | \ | \ |
| 139320 | *aflJ* | esterase | \ | \ | \ | \ | \ | \ |
| 139330 | *aflH* | short chain alcohol dehydrogenase | 0.15 | \ | \ | \ | \ | \ |
| 139340 | *aflS* | pathway regulator | \ | \ | \ | \ | \ | \ |
| 139360 | *aflR* | transcription activator | \ | \ | \ | \ | \ | \ |
| 139370 | *aflB* | fatty acid synthase beta subunit | \ | \ | \ | \ | \ | \ |
| 139380 | *aflA* | fatty acid synthase alpha subunit | \ | \ | \ | \ | \ | \ |
| 139390 | *aflD* | reductase | \ | \ | \ | \ | \ | \ |
| 139400 | *aflCa* | hypothetical protein | \ | \ | \ | \ | \ | \ |
| 139410 | *aflC* | polyketide synthase | 0.11 | 0.09 | 0.03 | 3.26 | 2.66 | 1.23 |
| 139420 | *aflT* | transmembrane protein | \ | \ | \ | \ | \ | \ |
| 139430 | *aflU* | P450 monooxygenase | \ | \ | \ | \ | \ | \ |
| 139440 | *aflF* | dehydrogenase | \ | \ | \ | \ | \ | \ |
| 046360 | *accA* | acetyl-CoA carboxylase putative | 0.84 | 0.87 | 0.18 | 4.61 | 4.78 | 0.97 |

\ means the proteins were not detected in this study. R, M, P and Y stand for rice, maize, peanut and YES media, respectively.

Table S5 Transcriptional changes of DEGs involved in carbon metabolism in maize vs peanut substrates

| Gene ID  (AFLA_xxx) | Gene function | log_2_Fold change  (M/P) | Pathway term |
| --- | --- | --- | --- |
| 020590 | aldose 1-epimerase putative | -4.27 | Glycolysis / Gluconeogenesis (AFV00010) |
| 031570 | pyruvate decarboxylase PdcA putative | 2.65 | Glycolysis / Gluconeogenesis (AFV00010) |
| 035290 | pyruvate dehydrogenase putative | 1.54 | Glycolysis / Gluconeogenesis (AFV00010) |
| 039690 | phosphoglycerate mutase putative | -1.02 | Glycolysis / Gluconeogenesis (AFV00010) |
| 042390 | glyceraldehyde 3-phosphate dehydrogenase putative | 3.01 | Glycolysis / Gluconeogenesis (AFV00010) |
| 091380 | phosphoglycerate mutase family protein | 1.10 | Glycolysis / Gluconeogenesis (AFV00010) |
| 015810 | citrate synthase putative | 1.11 | Citrate cycle (AFV00020) |
| 049290 | citrate synthase Cit1 putative | -1.81 | Citrate cycle (AFV00020) |
| 107660 | succinyl-CoA synthetase beta subunit putative | -1.09 | Citrate cycle (AFV00020) |
| 129510 | aconitase putative | 2.32 | Citrate cycle (AFV00020) |
| 128510 | 6-phosphogluconate dehydrogenase decarboxylating | 2.26 | Pentose phosphate pathway (AFV00030) |
| 022220 | 6-phosphofructo-2-kinase 1 | 1.06 | Fructose and mannose metabolism (AFV00051) |
| 040380 | mannitol dehydrogenase putative | 1.26 | Fructose and mannose metabolism (AFV00051) |
| 042010 | zinc-dependent alcohol dehydrogenase putative | -0.80 | Fructose and mannose metabolism (AFV00051) |
| 043980 | mannitol-1-phosphate dehydrogenase | 1.48 | Fructose and mannose metabolism (AFV00051) |
| 073650 | mandelate racemase lactonizing enzyme family protein | -2.84 | Fructose and mannose metabolism (AFV00051) |
| 094630 | triosephosphate isomerase | 1.10 | Fructose and mannose metabolism (AFV00051) |
| 107550 | alcohol dehydrogenase putative | -1.99 | Fructose and mannose metabolism (AFV00051) |
| 111260 | mannose-6-phosphate isomerase class I | 1.33 | Fructose and mannose metabolism (AFV00051) |
| 119950 | fructose-bisphosphate aldolase putative | -2.14 | Fructose and mannose metabolism (AFV00051) |
| 133950 | triosephosphate isomerase | 0.85 | Fructose and mannose metabolism (AFV00051) |
| 038640 | fatty acid synthase alpha subunit putative | 1.23 | Fatty acid biosynthesis (AFV00061) |
| 116820 | hypothetical protein | 1.11 | Fatty acid biosynthesis (AFV00061) |
| 116830 | hypothetical protein | 1.59 | Fatty acid biosynthesis (AFV00061) |
| 024290 | alcohol dehydrogenase putative | -1.16 | Fatty acid biosynthesis (AFV00061) |
| 048690 | alcohol dehydrogenase putative | 1.84 | Fatty acid biosynthesis (AFV00061) |
| 049020 | acyl-CoA dehydrogenase family protein | -1.30 | Fatty acid biosynthesis (AFV00061) |
| 077220 | NADPH flavin oxidoreductase putative | -1.34 | Fatty acid biosynthesis (AFV00061) |
| 085490 | P450 family fatty acid hydroxylase putative | 1.59 | Fatty acid biosynthesis (AFV00061) |
| 108790 | aldehyde dehydrogenase AldA putative | -0,90169 | Fatty acid biosynthesis (AFV00061) |
| 115890 | acyl-CoA oxidase putative | 1.27 | Fatty acid biosynthesis (AFV00061) |
| 125860 | alcohol dehydrogenase putative | -0.76 | Fatty acid biosynthesis (AFV00061) |
| 131960 | electron transport oxidoreductase putative | -1.69 | Fatty acid biosynthesis (AFV00061) |
| 002830 | alpha-alpha-trehalose-phosphate synthase subunit | 1.52 | Starch and sucrose metabolism (AFV00500) |
| 010980 | maltase MalT | -1.47 | Starch and sucrose metabolism (AFV00500) |
| 014190 | beta-glucosidase putative | -6.04 | Starch and sucrose metabolism (AFV00500) |
| 023350 | beta-glucosidase putative | -2.72 | Starch and sucrose metabolism (AFV00500) |
| 023490 | alpha-amylase putative | 2.45 | Starch and sucrose metabolism (AFV00500) |
| 026140 | alpha-amylase putative | 1.64 | Starch and sucrose metabolism (AFV00500) |
| 028260 | exo-beta-1-3-glucanase (Exg1) putative | -1.84 | Starch and sucrose metabolism (AFV00500) |
| 033200 | beta-glucosidase putative | -1.12 | Starch and sucrose metabolism (AFV00500) |
| 034950 | glucoamylase precursor putative | -1.61 | Starch and sucrose metabolism (AFV00500) |
| 051140 | beta-glucosidase putative | -3.15 | Starch and sucrose metabolism (AFV00500) |
| 057030 | beta-glucosidase | 1.10 | Starch and sucrose metabolism (AFV00500) |
| 073260 | hexokinase putative | -0.95 | Starch and sucrose metabolism (AFV00500) |
| 074250 | polygalacturonase precursor putative | 1.84 | Starch and sucrose metabolism (AFV00500) |
| 081340 | glycogen debranching enzyme Gdb1 putative | 0.84 | Starch and sucrose metabolism (AFV00500) |
| 083300 | alpha-glucosidase putative | -2.65 | Starch and sucrose metabolism (AFV00500) |
| 087630 | alpha-alpha-trehalose-phosphate synthase subunit | 1.69 | Starch and sucrose metabolism (AFV00500) |
| 097890 | Glycosyl hydrolases family 32 superfamily | -1.69 | Starch and sucrose metabolism (AFV00500) |
| 103730 | alpha-alpha-trehalose-phosphate synthase subunit Tps2 | 1.51 | Starch and sucrose metabolism (AFV00500) |
| 108160 | extracellular polygalacturonase putative | -3.63 | Starch and sucrose metabolism (AFV00500) |
| 122400 | glucan 1-4-alpha-glucosidase putative | -1.11 | Starch and sucrose metabolism (AFV00500) |
| 124740 | beta-galactosidase putative | -4.74 | Starch and sucrose metabolism (AFV00500) |
| 126780 | beta-glucosidase 2 precursor putative | -4.46 | Starch and sucrose metabolism (AFV00500) |
| 131370 | alpha-alpha-trehalose phosphate synthase subunit TPS3 | 1.14 | Starch and sucrose metabolism (AFV00500) |
| 003700 | mitochondrial cytochrome b putative | 1.33 | Pyruvate metabolism (AFV00620) |
| 031910 | acetate kinase putative | 1.53 | Pyruvate metabolism (AFV00620) |
| 035290 | pyruvate dehydrogenase putative | 1.54 | Pyruvate metabolism (AFV00620) |
| 112920 | mitochondrial cytochrome b2-like putative | -1.67 | Pyruvate metabolism (AFV00620) |
| 136660 | lactoylglutathione lyase (Glo1) putative | -0.91 | Pyruvate metabolism (AFV00620) |

M stands for maize substrate and P stands for peanut substrate.

Table S6 Transcriptional changes of genes involved in growth, development, stress adaption, and signal transduction in maize substrate vs peanut substrate.

| Gene ID  (AFLA_xxx) | Gene name | log_2_Fold Change  (M/P) | *p* value | significant | Gene description |
| --- | --- | --- | --- | --- | --- |
| 066460 | *veA* | 1.93 | 0.0029256 | TRUE | developmental regulator AflYf / VeA |
| 033290 | *laeA* | 0.10 | 0.77957 | FALSE | regulator of secondary metabolism LaeA |
| 084190 | *velB* | -0.06 | 0.85061 | FALSE | conserved hypothetical protein |
| 083380 | *pbsB* | 0.08 | 0.8739 | FALSE | MAP kinase kinase (Pbs2) putative |
| 062500 | *maf1* | 0.00 | 0.95449 | FALSE | mitogen-activated protein kinase MAF1 |
| 103480 | *ste7* | 0.19 | 0.61704 | FALSE | MAP kinase kinase Ste7 |
| 035530 | *ste20* | -0.07 | 0.80835 | FALSE | serine/threonine kinase Ste20 |
| 048880 | *ste11* | 0.04 | 0.85574 | FALSE | MAP kinase kinase kinase Ste11 |
| 052570 | *mpkA* | 0.09 | 0.88627 | FALSE | MAP kinase MpkA |
| 051240 | *map2k* | -0.06 | 0.79961 | FALSE | MAP kinase kinase (Mkk2) putative |
| 034170 | *fus3* | 0.26 | 0.3962 | FALSE | MAP kinase FUS3/KSS1 |
| 031560 | *bck1* | 1.30 | 0.00017045 | TRUE | MAP kinase kinase kinase (Bck1), putative |
| 068590 | *sskB* | 0.54 | 0.067757 | FALSE | MAP kinase kinase kinase SskB putative |
| 061090 | *sakA1* | 0.14 | 0.94176 | FALSE | MAP kinase SakA |
| 099500 | *sakA2* | 0.91 | 0.064666 | FALSE | MAP kinase SakA |
| 031340 | *atfA* | 0.10 | 0.70583 | FALSE | bZIP transcription factor (AtfA), putative |
| 094010 | *atfB* | 0.88 | 0.0095637 | FALSE | bZIP transcription factor (Atf21) putative |
| 129340 | *ap-1* | 0.05 | 0.92482 | FALSE | conserved hypothetical protein |
| 110650 | *msnA* | -0.06 | 0.88947 | FALSE | C2H2 transcription factor (Seb1) putative |
| 091490 | *mtfA* | -0.47 | 0.11259 | FALSE | C2H2 finger domain protein putative |
| 034540 | *srrA* | 0.08 | 0.87847 | FALSE | stress response transcription factor SrrA/Skn7, putative |
| 062210 | *sskA* | 0.19 | 0.56804 | FALSE | response regulator putative |
| 026790 | *ppoA* | -0.76 | 0.016997 | FALSE | fatty acid oxygenase PpoA, putative |
| 120760 | *ppoB* | -2.99 | 0.0000548 | TRUE | fatty acid oxygenase, putative |
| 030430 | *ppoC* | -0.11 | 0.94635 | FALSE | conserved hypothetical protein |
| 101920 | *fluG* | -0.80 | 0.011169 | FALSE | extracellular developmental signal biosynthesis protein FluG |
| 002850 | *AfPXG* | 1.94 | 8.47E-08 | TRUE | calcium binding protein Caleosin, putative |
| 025100 | *gpdA* | 0.79 | 0.11377 | FALSE | glyceraldehyde 3-phosphate dehydrogenase GpdA |
| 046760 | *gfdB* | 1.09 | 0.10008 | FALSE | glycerol 3-phosphate dehydrogenase (GfdB) putative |
| 060740 | *gprA* | -0.79 | 0.014159 | FALSE | mating-type alpha-pheromone receptor PreB |
| 061620 | *gprB* | 0.25 | 0.46637 | FALSE | a-pheromone receptor PreA |
| 074150 | *gprC* | -1.58 | 0.0000015 | TRUE | conserved hypothetical protein |
| 135680 | *gprD* | -0.70 | 0.17637 | FALSE | G protein-coupled receptor GprD |
| 006880 | *gprF* | -0.11 | 0.66711 | FALSE | PQ loop repeat protein |
| 067770 | *gprG* | 0.95 | 0.0026451 | TRUE | PQ loop repeat protein |
| 006920 | *gprH* | -2.28 | 6.4E-12 | TRUE | cAMP receptor-like protein, putative |
| 127870 | *gprJ* | 1.12 | 0.00028576 | TRUE | vacuolar membrane PQ loop repeat protein |
| 009790 | *gprK* | 0.04 | 0.73158 | FALSE | conserved hypothetical protein |
| 075000 | *gprM* | -2.56 | 5.39E-15 | TRUE | conserved hypothetical protein |
| 032130 | *gprO* | -0.22 | 0.4582 | FALSE | hemolysin-III channel protein Izh2 putative |
| 088190 | *gprP* | -0.59 | 0.054595 | FALSE | IZH family channel protein (Izh3) putative |
| 023070 | *gprR* | -1.22 | 0.00014932 | TRUE | integral membrane protein |
| 006320 | *gprS* | 0.01 | 0.92018 | FALSE | PQ loop repeat protein |
| 018340 | *fadA* | 0.23 | 0.52441 | FALSE | G-protein complex alpha subunit GpaA/FadA |
| 093240 | *sfaD* | -0.13 | 0.63414 | FALSE | G-protein complex beta subunit SfaD |
| 032870 | *pkaR* | -0.30 | 0.26491 | FALSE | cAMP-dependent protein kinase regulatory subunit PkaR |
| 135040 | *pkaC* | 0.87 | 0.1325 | FALSE | cAMP-dependent protein kinase catalytic subunit PkaC1 |
| 018930 | *capA* | 0.05 | 0.85945 | FALSE | adenylyl cyclase-associated protein (cap) |
| 071410 | *somA* | 0.39 | 0.24681 | FALSE | cAMP-dependent protein kinase pathway protein Som1 |
| 112560 | *sok1* | -0.47 | 0.11456 | FALSE | cAMP-mediated signaling protein Sok1 putative |
| 134680 | *creA* | 0.37 | 0.25056 | FALSE | C2H2 transcription factor (CreA) putative |
| 062250 | *snf1* | 2.4898 | 0.0023792 | TRUE | carbon catabolite derepressing protein kinase Snf1 putative |
| 049870 | *areA* | 0.79 | 0.014291 | FALSE | GATA transcriptional activator AreA |
| 030580 | *pacC* | -0.32 | 0.19882 | FALSE | C2H2 transcription factor PacC putative |
| 012010 | *farB* | -1.48 | 0.00000078 | TRUE | C6 transcription factor (Ctf1B) putative |

M stands for maize substrate and P stands for peanut substrate. As *p*<0.05, it was considered as the significant difference.

Table S7 The primers for RT-qPCR analyses

| Gene name | Gene ID  (AFLA_xxx) | Primer | Fragment length (bp) |
| --- | --- | --- | --- |
| *aflA* | 139380 | F: aattgctcaacttcctacc | 143 |
|  |  | R: ttgggttggcctcgccaaag |  |
| *aflB* | 139370 | F: atcttcccctgctgcaatag | 152 |
|  |  | R: aatagagcgctaggctaagtaac |  |
| *aflC* | 139410 | F: ttcgaaccgctctagtgtgcc | 140 |
|  |  | R: agcgactgcagcttaccgcc |  |
| *aflK* | 139190 | F: tgtcccaggatggaactgcttc | 152 |
|  |  | R: ttgataccagtcaaatagagg |  |
| *aflH* | 139330 | F: tgcatcaggcatcgggttgg | 145 |
|  |  | R: tcgcagcagacgtagtggacg |  |
| *aflO* | 139220 | F: agcagtggacccctacaag | 151 |
|  |  | R: tccgaagaatgcgaccaagg |  |
| *aflV* | 139180 | F: taaggtccctggtccctgg | 151 |
|  |  | R: atcggctatgtcaacctcc |  |
| *aflR* | 139360 | F: aggagaaaccggcctgtgctcg | 158 |
|  |  | R: aagtccctgttccgacctgg |  |
| *aflS* | 139340 | F: tgaccatctccgacccgttc | 154 |
|  |  | R: acgccagcacctggaactcc |  |
| *accA* | 046360 | F: agaagatttgcgggcaaatgc | 149 |
|  |  | R: atccggcccaaaccgcgtgg |  |
