# Supplementary material for: Comprehensive Transcriptome and Proteome Analyses Reveal the Modulation of Aflatoxin Production by Aspergillus flavus on Different Crop Substrates
Source: Front Microbiol. 2020 Jul 14;11:1497. doi: 10.3389/fmicb.2020.01497 (PMC7371938; doi:10.3389/fmicb.2020.01497)
Supplement: Supplementary file 2 [file Data_Sheet_2.doc]

**Fig. S1 The scatter diagrams of KEGG enriched results.**

The differential expressions *A. flavus* genes in different media were analyzed by transcriptome, and were enriched by KEGG pathway analyses. Top 20 enriched pathways showed in this graph. The rich factors and the pathway names showed in X-axis and Y-axis, respectively. The size of spots represented the number of enriched genes, and the different colors described the *q*-value. (A) The comparison of maize media versus YES media. (B) The comparison of rice media versus YES media. (C) The comparison of peanut media versus YES media. (D) The comparison of maize media versus peanut media. (E) The comparison of rice media versus peanut media. (F) The comparison of maize media versus rice media.

**Fig. S2 The bar charts of GO enriched results.**

The differentially expressed genes of *A. flavus* from different media were analyzed by transcriptome, and were enriched by GO term analyses. The number of enriched genes and the names of GO terms showed in X-axis and Y-axis, respectively. The biological process, cellular components, and molecular function were represented by the green bars, orange bars, and blue bars, respectively. (A) The comparison of maize media versus YES media. (B) The comparison of rice media versus YES media. (C) The comparison of peanut media versus YES media. (D) The comparison of maize media versus peanut media. (E) The comparison of rice media versus peanut media. (F) The comparison of maize media versus rice media.

**Fig. S3 The bar charts of KEGG pathways enriched analyse in the intracellular proteome.**

The differentially expressed intracellular proteins of *A. flavus* from different media were analyzed by proteome, and were enriched by KEGG pathway analyses. Top 20 enriched pathways displayed in this graph. The numbers of enriched proteins showed the top of bar. (A) The comparison of maize media versus YES media. (B) The comparison of rice media versus YES media. (C) The comparison of peanut media versus YES media. (D) The comparison of maize media versus peanut media. (E) The comparison of rice media versus peanut media. (F) The comparison of maize media versus rice media.

**Fig. S4** **The bar charts of GO enriched analyse in the intracellular proteome.**

The differentially expressed intracellular proteins of *A. flavus* from different media were analyzed by proteome, and were enriched by GO term analyses. The number of enriched proteins and the names of GO terms showed in X-axis and Y-axis, respectively. The biological process, molecular function, and cellular components were represented by the red bars, blue bars, and orange bars, respectively.(A) The comparison of maize media versus YES media. (B) The comparison of rice media versus YES media. (C) The comparison of peanut media versus YES media. (D) The comparison of maize media versus peanut media. (E) The comparison of rice media versus peanut media. (F) The comparison of maize media versus rice media.

**Fig. S5 GO annotation and KEGG pathways enrichment of differential expressed extracellular proteins in three crop substrates.**

The differentially expressed extracellular proteins of *A. flavus* from different media were analyzed by proteome, and were enriched by GO term and KEGG pathway analyses, respectively. The KEGG enriched results displayed with the bar charts and the numbers of enriched proteins showed the top of bar. (A) The top 20 KEGG enriched pathways in comparison of maize media versus peanut media. (B) The top 20 KEGG enriched pathways in comparison of rice media versus peanut media. (C) The top 20 KEGG enriched pathways in comparison of maize media versus rice media. The GO enriched results also showed with bar charts in this figure, with red bars representing biological process, blue bars representing molecular function, and orange bars representing cellular components. (D) The GO enriched analyses in comparison of maize media versus peanut media. (E) The GO enriched analyses in comparison of rice media versus peanut media. (F) The GO enriched analyses in comparison of maize media versus rice media.
