# Supplementary figures and images for: Comprehensive Transcriptome and Proteome Analyses Reveal the Modulation of Aflatoxin Production by Aspergillus flavus on Different Crop Substrates
Source: Front Microbiol. 2020 Jul 14;11:1497. doi: 10.3389/fmicb.2020.01497 (PMC7371938; doi:10.3389/fmicb.2020.01497)

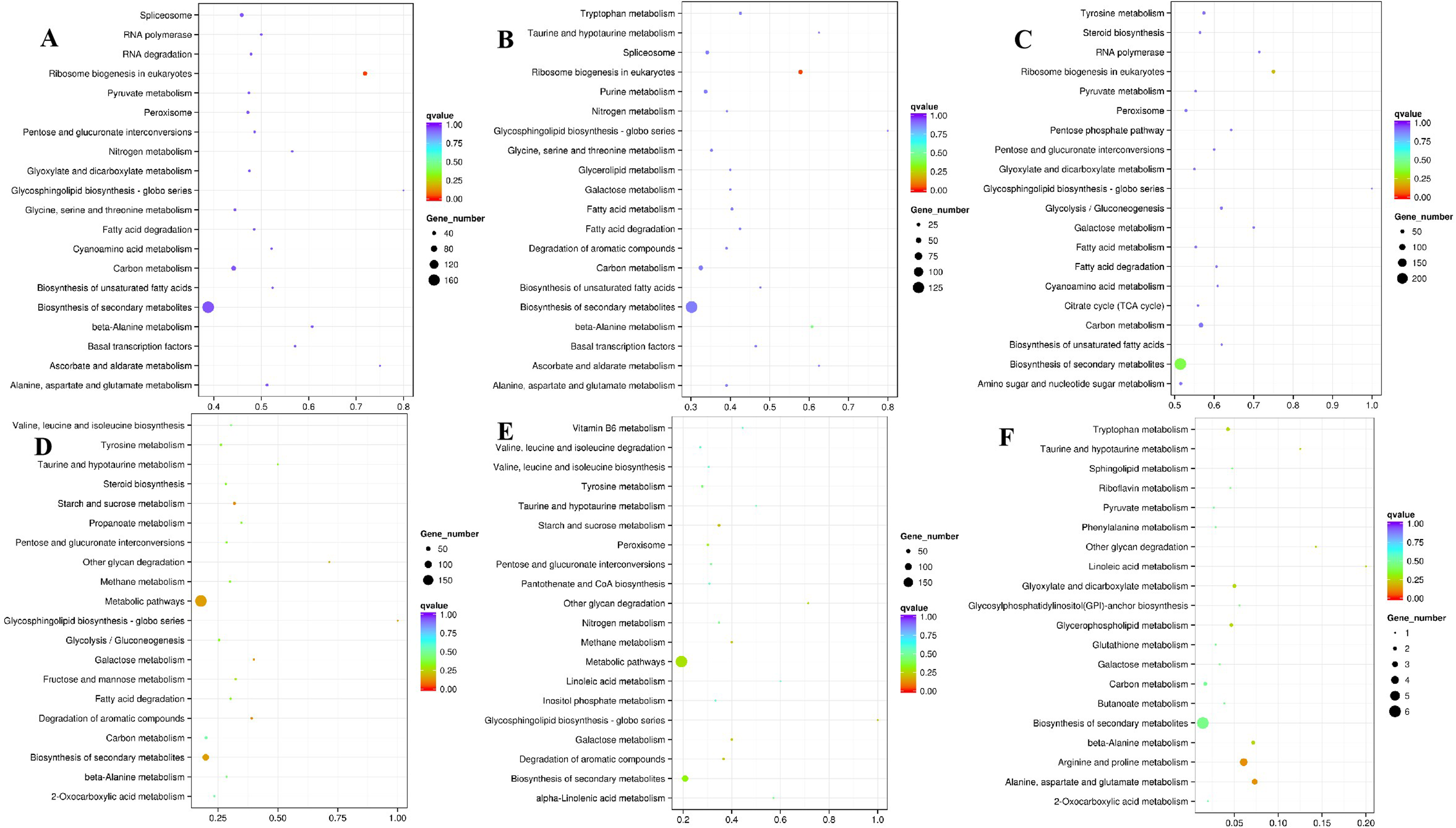

Supplement: Supplementary file 3 [file Image_1.TIF]

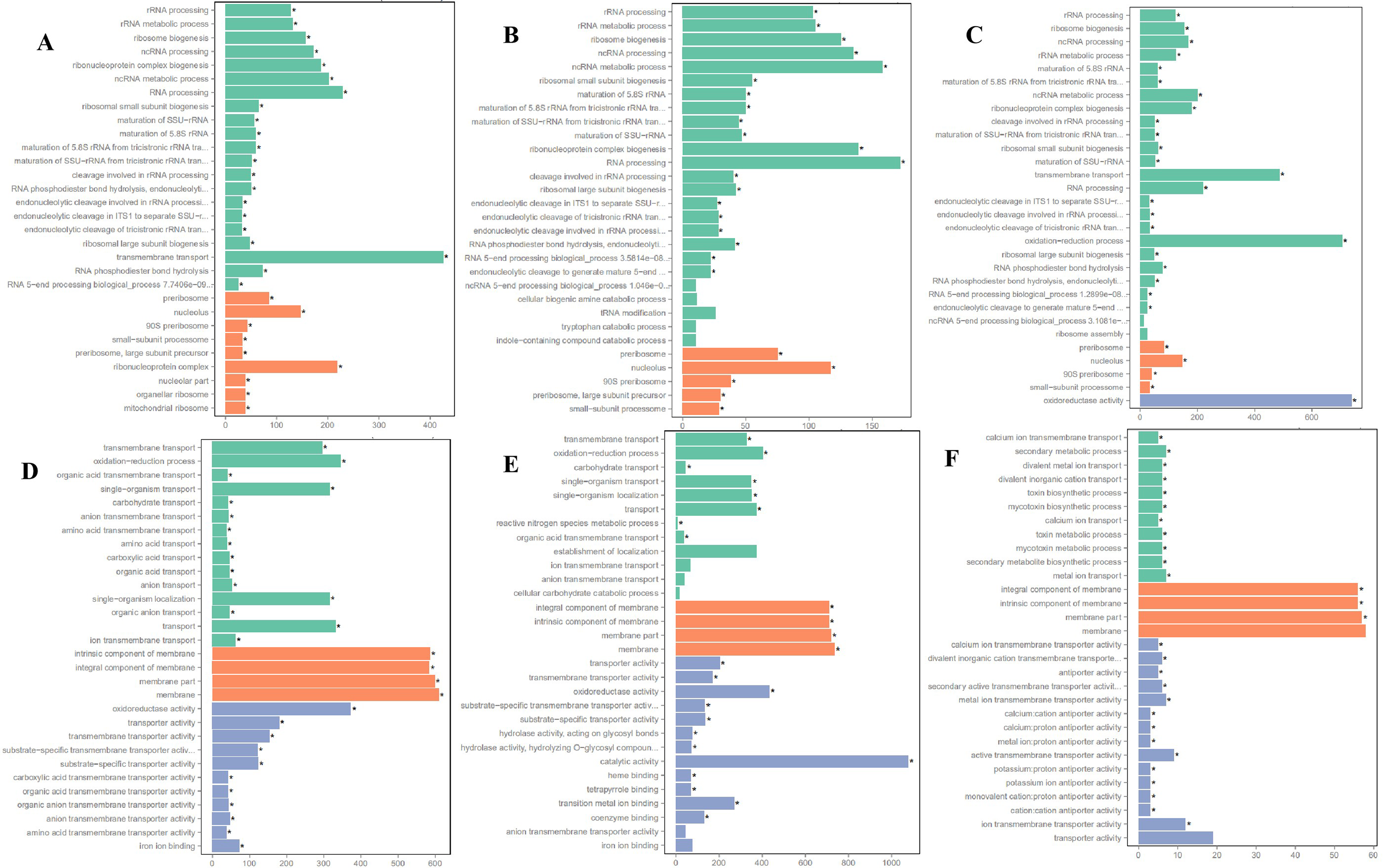

Supplement: Supplementary file 4 [file Image_2.TIF]

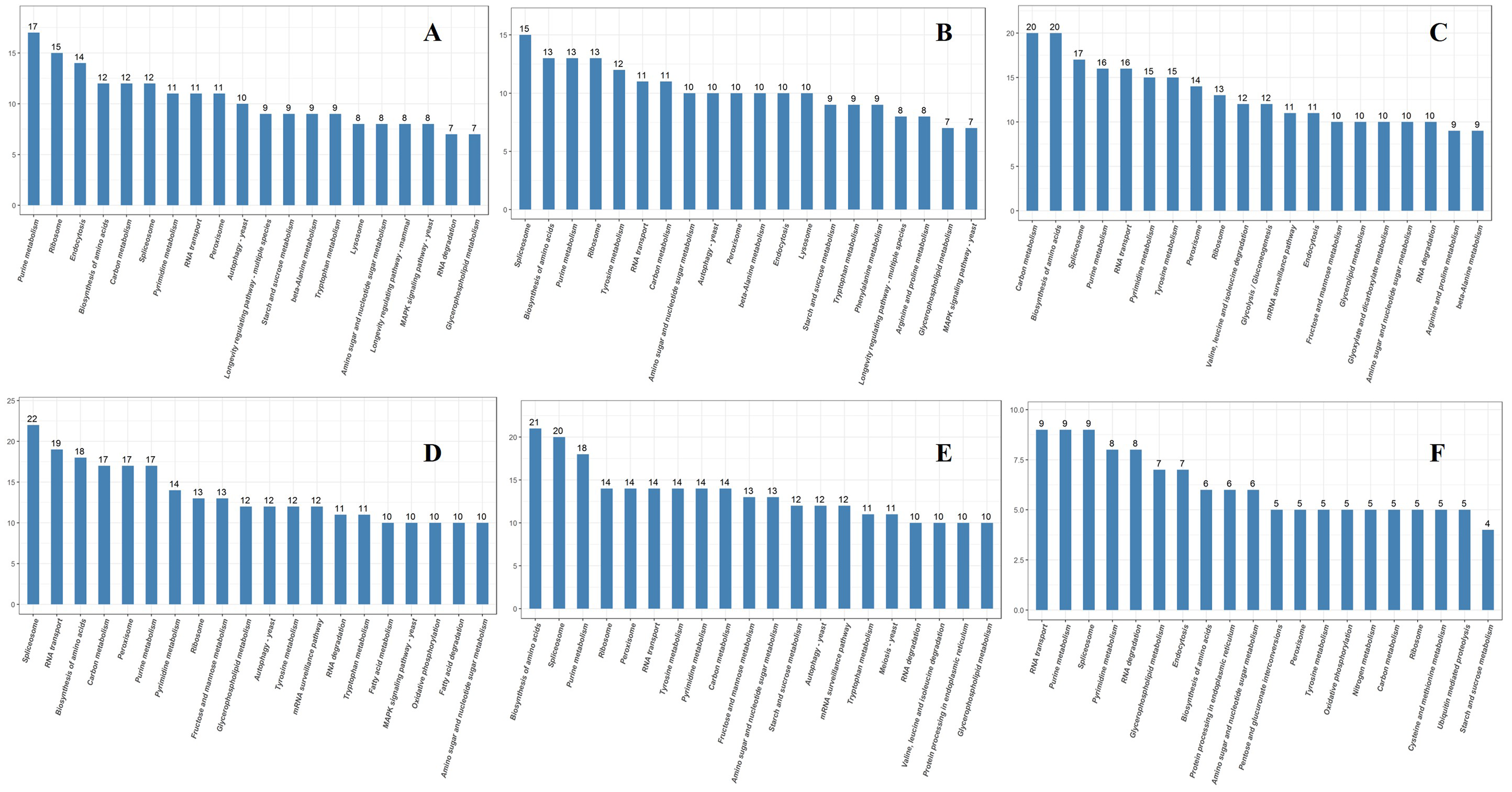

Supplement: Supplementary file 5 [file Image_3.TIF]

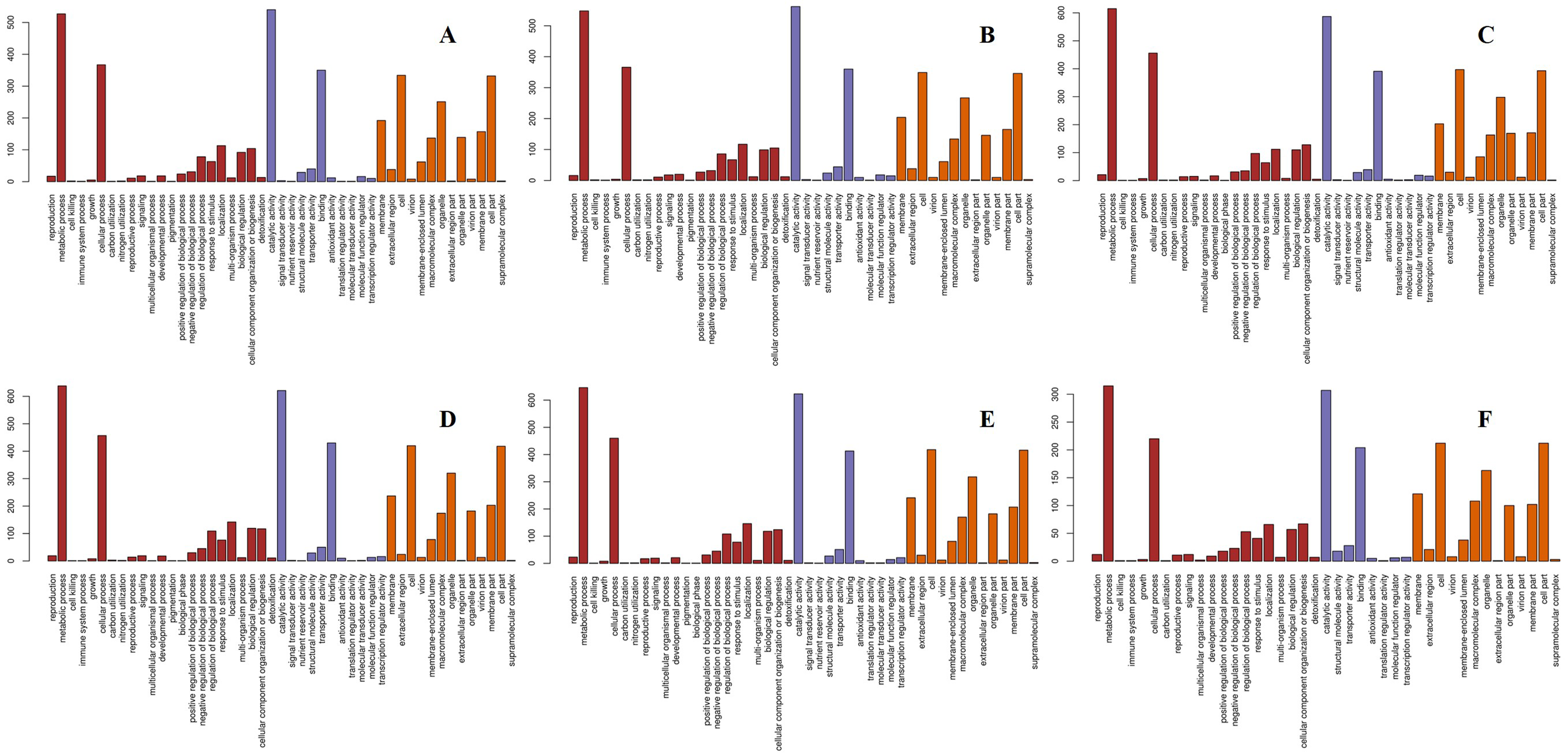

Supplement: Supplementary file 6 [file Image_4.TIF]

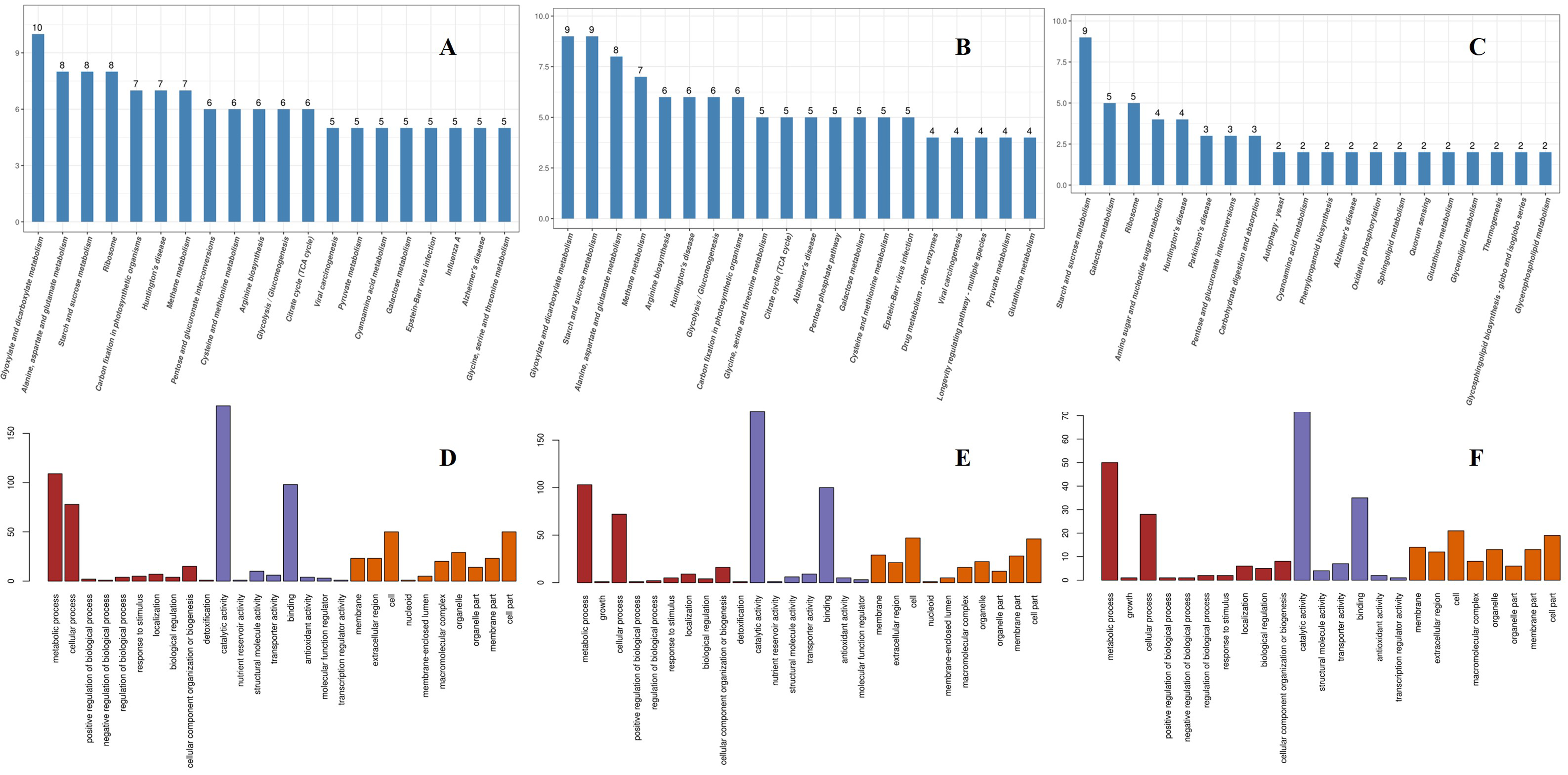

Supplement: Supplementary file 7 [file Image_5.TIF]
